# Multi-Omics Analysis Identifies Fibroblast-Associated Stromal–Immune Networks Linked to Pregnancy-Associated Attenuation of Imiquimod-Induced Psoriatic Dermatitis

**DOI:** 10.3390/biomedicines14092048

**Published:** 2026-09-11

**Authors:** Jiaqing Shen, Lisha Song, Zihan Ye, Mingjun Jiang, Yi Liu

**Affiliations:** Hospital for Skin Diseases, Institute of Dermatology, Chinese Academy of Medical Sciences & Peking Union Medical College, Nanjing 210042, China; pyssjqmay@student.pumc.edu.cn (J.S.); s2023017016@student.pumc.edu.cn (L.S.); s2024017019@student.pumc.edu.cn (Z.Y.)

**Keywords:** psoriasis, pregnancy, fibroblasts, single-cell RNA sequencing, Gas6, progranulin

## Abstract

**Background:** Psoriasis is a chronic, immune-mediated inflammatory skin disorder that frequently exhibits spontaneous clinical remission during pregnancy. However, the underlying cell-type-specific transcriptomic alterations—particularly the involvement of dermal fibroblasts and hormone receptor signaling—remain poorly understood. **Methods:** We established an imiquimod (IMQ)-induced psoriasis-like dermatitis model in BALB/c mice comparing pregnant and non-pregnant cohorts. Multi-omics profiling combing bulk and single-cell RNA sequencing was integrated with computational intercellular communication and trajectory analysis. In vitro hormone-stimulation assays with ELISA quantification and in situ multiplex immunofluorescence were performed for experimental evaluation. **Results:** Compared with non-pregnant IMQ-treated mice, pregnant mice exhibited reduced psoriasis area and severity index scores and diminished epidermal thickening. Bulk RNA sequencing revealed downregulated Th1/Th17 inflammatory transcripts and upregulated hormone-associated genes (e.g., *Pgr*, *Esr1*, *Ghr*). Single-cell RNA sequencing identified 11 distinct cell types, with fibroblasts displaying distinct hormone receptor profiles and functional heterogeneity. Fibroblasts displayed transcripts encoding both anti-inflammatory factors (e.g., *Tgfb2*, *Gas6*, *Grn*) and pro-inflammatory factors (e.g., *Fgf7*, *Mif*). Keratinocytes exhibited an anti-inflammatory transcriptional phenotype with attenuated proliferation signatures. Monocytes/macrophages shifted toward M2-like transcriptional signatures and displayed computationally predicted communication with fibroblasts via candidate pathways including GRN and Gas6. Experimentally, primary mouse dermal fibroblasts stimulated with estradiol, progesterone, and growth hormone in vitro significantly increased secretion of PGRN. Gas6 secretion was significantly augmented by estradiol and growth hormone, whereas progesterone induced a modest elevation with borderline significance (*p* = 0.060). In contrast, TGF-β2 protein remained undetectable in culture supernatants. Furthermore, multiplex immunofluorescence demonstrated significantly increased expression of the Gas6 receptor MerTK on dermal macrophages in the IMQ + pregnancy group. **Conclusions:** Pregnancy-induced hormonal fluctuations may be associated with the amelioration of IMQ-induced psoriatic dermatitis, which correlates with a fibroblast-involved immunoregulatory network. These findings highlight the association of stromal-immune crosstalk in disease remission and suggest the GRN and Gas6 pathways as candidate pathways for future functional validation.

## 1. Introduction

Psoriasis is a chronic, immune-related skin disease characterized by hyperproliferation of keratinocytes and cutaneous infiltration of immune cells including neutrophils, lymphocytes and monocytes, leading to circumscribed, scaling and erythematous plaques [1]. Although both genders are susceptible to psoriasis, recent research has highlighted the profound impact of changes in hormone levels on disease manifestation and progression in women [2,3]. As a pivotal physiological stage in a woman’s life, pregnancy induces substantial hormone shifts, with extraordinarily high levels of estrogen and progesterone to maintain the pregnancy and promote fetal development, which can profoundly influence psoriasis symptoms [4]. According to the research, up to 60% of pregnant women with psoriasis may see an improvement or even achieve remission, while 26.4% of female psoriasis patients exhibit disease exacerbation during pregnancy, and more than half of patients experienced worsening in the postpartum period [4,5,6].

Current studies have illustrated the anti-inflammatory and immunosuppressive effects of estrogen and progesterone on psoriasis. Estrogen maintains the balance of T helper (Th) cell subsets and regulatory T cells (Tregs) by transferring immune response from the Th1 and Th17 profile to the more anti-inflammatory Th2 profile [2,7]. Additionally, estrogen promotes the secretion of anti-inflammatory cytokines, including interleukin-10 (IL-10) and transforming growth factor-beta (TGF-β), which can sustain the immune tolerance environment [2,8,9,10]. It also downregulates production of pro-inflammatory cytokines, such as tumor necrosis factor-alpha (TNF-α), interleukin-6 (IL-6), and interferon-gamma (IFN-γ), by affecting immune cells like T cells, B cells, dendritic cells, and macrophages [2,11]. Notably, previous work by Adachi et al. established that 17β-estradiol directly suppresses IMQ-induced psoriatic inflammation by restraining neutrophils and inflammatory macrophages, thereby dampening IL-1β and IL-17A production [12]. Progesterone has similar effects on immune homeostasis by modulating CD4+ T cells. Despite these findings, how pregnancy affects psoriasis in vivo remains unclear [13].

Fibroblasts are a highly abundant cell type in the skin that have been generally overlooked in skin immune environment until recently. Recent studies have proved the important role of fibroblasts in psoriasis by expression of several cytokines, chemokines and antimicrobials [14,15,16]. Fibroblasts also affect psoriasis directly through the Th1/Th17 axis due to the expression of TNF receptor 1 [17]. Beyond their pro-inflammatory potential, fibroblasts play a significant role in immune homeostasis. By the expression of cytokines like TGF-β and Growth Arrest Specific 6 (Gas6), fibroblasts promote the polarization of macrophages towards the M2 phenotype [18,19]. Also, fibroblasts can affect the proliferation of keratinocytes through the fibroblast growth factor (FGF) family [20]. However, few studies explore changes in fibroblasts in the skin during pregnancy.

In this study, we performed an unbiased comparison of total skin cells extracted from imiquimod-induced BALB/c psoriatic mice with or without pregnancy, using single-cell RNA sequencing (scRNA-seq) and bulk RNA sequencing (bulk RNA-seq). These unprecedented data uncovered the transcriptional landscape of the psoriatic dermatitis lesion during pregnancy, and emphasized specialized transcriptomic profiles and phenotypic heterogeneity of fibroblasts.

## 2. Materials and Methods

### 2.1. Experimental Animals and Establishment of an Imiquimod (IMQ)-Induced Skin Inflammation Model

Male and female BALB/c mice were obtained from GemPharmatech Co., Ltd. (Nanjing, China). Mice were housed in specific pathogen-free (SPF) facilities with controlled temperature (22 ± 1 °C), humidity (50 ± 5%), and 12 h light/dark cycles, with ad libitum access to food and water. All procedures were approved by the Institutional Animal Care and Use Committee at the Hospital for Skin Diseases, Institute of Dermatology, Chinese Academy of Medical Sciences & Peking Union Medical College, Nanjing, China (approval number 2024-DW-010), and complied with relevant guidelines and regulations. All methods are reported in accordance with the ARRIVE guidelines.

At the study onset, age-matched female BALB/c mice were randomized into three initial groups (vehicle, IMQ, and IMQ + pregnancy; n = 6 per group). Mice in the IMQ + pregnancy group were then co-housed with males (1:2 ratio) for mating, with the detection of a vaginal plug defining embryonic day 0 (E0). To account for asynchronous natural mating and eliminate potential batch effects, the following strategy was used: as each pregnant mouse reached E8, age-matched non-pregnant controls from the pre-randomized Vehicle and IMQ cohorts were simultaneously selected randomly on the same day to initiate the next procedure. Mice were anesthetized with the intraperitoneal injection of 1% pentobarbital sodium (40 mg/kg), and the dorsal hair was shaved off to expose the skin. From E10 onwards, the IMQ + pregnancy and IMQ groups were topically administered 62.5 mg of 5% IMQ cream (Aldara^®^; iNova Pharmaceuticals, Sydney, Australia) once daily for 5 consecutive days as previous described [21]. In contrast, the vehicle group was topically applied an equal volume of petrolatum as a vehicle control. All mice were euthanized by intraperitoneal injection of an overdose of pentobarbital sodium (≥150 mg/kg), in accordance with the recommendations of the American Veterinary Medical Association (AVMA) Guidelines for the Euthanasia of Animals (2020) [22] and institutional ethical standards, on E15 (i.e., the 6th day after the initiation of treatment), with consistent timing implemented across the non-pregnant groups. Dorsal skin tissue samples were promptly harvested for subsequent experimental analysis. From each initial cohort of six mice evaluated for in vivo phenotypic and histological parameters (n = 6 per group), three animals per group were randomly selected using a computer-generated random number sequence for downstream high-throughput transcriptome sequencing analysis (n = 3 per group). To maximize cross-platform biological concordance, the exact same three animals per group selected via computer randomization were used for both bulk RNA-seq and scRNA-seq.

Throughout the experimental period, digital photographs of the dorsal skin of mice in each group were captured daily at a fixed time to dynamically monitor changes in skin appearance. The severity of psoriasis-like lesions was quantitatively evaluated using the mouse psoriasis area and severity index (mPASI), which assesses three key indicators: erythema, scales, and skin thickness. Each indicator was scored on a 0–4 scale (0 = absent, 1 = mild, 2 = moderate, 3 = marked, 4 = severe), with a total mPASI score ranging from 0 to 12 points. The mPASI scoring was conducted by two independent blinded assessors.

### 2.2. Histological Analysis

Hematoxylin and eosin (H&E) staining was used to assess histopathological changes in dorsal lesional skin tissues described above. Samples were fixed in 10% neutral-buffered formalin for 24 h, embedded in paraffin, and sectioned into 4 µm slices. Sections were deparaffinized, rehydrated, stained with hematoxylin (5 min) and eosin (2 min), dehydrated through graded ethanol, and cleared in xylene. Coverslips were mounted for preservation, and images were captured using a light microscope (Olympus BX53, Olympus, Tokyo, Japan) at 100× magnification. For each mouse (n = 6 per group), the epidermal thickness was calculated by averaging measurements across 5 randomly selected high-power fields (400× magnification) per tissue section by two blinded assessors.

### 2.3. Bulk RNA Sequencing and Differentially Expressed Gene Analysis

Dorsal lesional skin tissues were harvested from the IMQ + pregnancy group (n = 3) and IMQ group (n = 3) as described above. Total RNA was extracted using the TRIzol reagent (Invitrogen, Carlsbad, CA, USA) according to the manufacturer’s protocol. RNA purity and quantification were evaluated using the NanoDrop 2000 spectrophotometer (Thermo Scientific, Waltham, MA, USA). RNA integrity was assessed using the Agilent 2100 Bioanalyzer (Agilent Technologies, Santa Clara, CA, USA). Then, the libraries were constructed using a VAHTS Universal V10 RNA-seq Library Prep Kit (Premixed Version, Vazyme, Nanjing, China) according to the manufacturer’s instructions. The transcriptome sequencing was conducted by OE Biotech Co., Ltd. (Shanghai, China).

The libraries were sequenced on an Illumina Novaseq 6000 platform, and 150 bp paired-end reads were generated. About 24 million raw reads for each sample were generated. Raw reads of FASTQ format were initially processed using fastp [23], and the low-quality reads were removed to obtain the clean reads. Then, about 23 million clean reads for each sample were retained for subsequent analyses. The clean reads were mapped to the reference genome using HISAT2 [24]. FPKM [25] of each gene was calculated, and the read counts of each gene were obtained by HTSeq-count [26]. Principal component analysis (PCA) was performed using R (v 4.3.3) to evaluate the biological duplication of samples.

After filtration and alignment, bulk RNA sequencing data were used for normalization, and differentially expressed genes (DEGs) were analyzed using the DESeq2 (v 1.42.1) software package. The statistical significance was calculated, and the adjusted *p* value is measured using the Benjamini–Hochberg false discovery rate (FDR) as the approach for multiple test correction [27]. To identify the differentially expressed genes, the adjusted *p*-value < 0.05 and |log_2_FC| > log_2_1.5 were set as the criteria. log_2_FC > log_2_1.5 means up-regulated genes and log_2_FC < −log_2_1.5 means downregulated genes.

### 2.4. Single-Cell Suspension Preparation

Fresh dorsal lesional skin tissues were harvested from the IMQ + pregnancy group (n = 3) and IMQ group (n = 3) as described above and quickly stored in Tissue Storage Solution (cat. 130-100-008; Miltenyi Biotec, Bergisch Gladbach, Germany) after euthanasia, then washed thrice with Hanks Balanced Salt Solution before digestion. The separated epidermis was digested into single-cell suspensions using trypsin-EDTA, while the dermis was mechanically disrupted and treated with 2 mg/mL Collagenase IV in RPMI 1640 for 1 h at 37 °C before filtration through 70 µm strainers. The combined cell suspensions from the epidermis and dermis were then treated with a kit (cat. 130-090-101; Miltenyi Biotec) to enhance cell viability for subsequent single-cell sequencing.

### 2.5. Single-Cell RNA-Seq Protocol and Data Analysis

Single cells were encapsulated in droplets using 10× Genomics GemCode Technology and processed according to the manufacturer’s instructions. Briefly, every cell and every transcript was uniquely barcoded using a unique molecular identifier (UMI). Libraries were generated and sequenced from the cDNA, and the 10× barcodes were used to link individual reads back to the individual partitions. The single-cell 3′ protocol produces Illumina-ready sequencing libraries. The FASTQ files were processed and aligned to the GRCm39 mouse reference genome using CellRanger software (v 8.0.1) from 10× Genomics, with unique UMI counts summarized for each barcode. The sequencing was provided by OE Biotech Co., Ltd. (Shanghai, China).

### 2.6. Quality Control Metrics and Data Processing

For downstream analyses after initial Cell Ranger metric assessment, low-quality cells were removed to eliminate cell-specific biases. We used R package scDblFinder (v 1.16.0) to remove the doublets (two different cell types within a single droplet) from the dataset at an expected doublet rate of 10%. We used R package Seurat (v 5.0.3) [28] to further enhance the control of data quality in the following ways: cells with fewer than 200 genes or more than 6500 genes detected, and cells for which more than 10% of UMIs were derived from mitochondrial genes were excluded. Post-quality control, 57,056 cells remained for downstream bioinformatics analyses. Detailed per-sample post-quality-control metrics including cell numbers after QC, median genes detected per cell, median UMI counts, and doublet-removal statistics are documented in Table S1. The normalization was performed with the ‘NormalizeData’ function, and data scaling with the ‘ScaleData’ function. Subsequently, principal component analysis was conducted using the top 2000 most variable genes. Data were integrated by Canonical Correlation Analysis (CCA) with default settings. The top 50 principal components (PCs) were utilized for Uniform Manifold Approximation and Projection (UMAP). Unsupervised clustering was undertaken with a resolution of 0.3. Cell types were annotated based on the expression of canonical marker genes and PanglaoDB.

### 2.7. Analysis of Differentially Expressed Genes in the scRNA-Seq Dataset

DEGs and cluster-specific genes were identified using the ‘FindMarkers’ function in the ‘Seurat’ R package using the Mann–Whitney U-test. We identified differentially expressed genes based on the following criteria: |logFC| > 0.5 and adjusted *p*-value < 0.05. Given that individual cells derived from the same animal do not constitute completely independent biological replicates, these cell-level DEG results were treated as strictly exploratory. Heatmaps of DEGs for fibroblasts, keratinocytes and monocytes/macrophages are shown in Figure S9 to illustrate the per-animal consistency.

### 2.8. Gene Set Enrichment Analysis

For DEGs and cluster-specific genes in the bulk RNA-seq dataset and scRNA-seq dataset, R package clusterProfiler (v 4.10.1) was used to conduct functional annotation [29,30]. Among them, Gene Ontology (GO) enrichment analysis, Kyoto Encyclopedia of Genes and Genomes (KEGG) enrichment analysis, and gene set enrichment analysis (GSEA) were performed using ‘enrichGO’ and ‘enrichKEGG’, ‘gseKEGG’, and ‘gseGO’ functions, with terms significant at *p*-value < 0.05.

### 2.9. Cell–Cell Interaction Analysis

The cell–cell interactions between different cell types were evaluated via the R package CellChat (v 1.6.1) [31,32]. CellChat was executed on the normalized data matrix from the Seurat object after quality control before integration, and used the ‘CellChatDB.mouse’ database curated in CellChat. The minimum cell-number threshold for each cell type population was set to 10 cells, and variations in cell-type abundance across samples were statistically considered using the default set of the “liftCellChat” function. All resulting communication paths represent predicted ligand–receptor interactions and predicted information flows rather than directly measured protein-level signaling.

### 2.10. Trajectory Analysis

To investigate the potential cell differentiation trajectory of fibroblasts and monocytes/macrophages, we used the “Monocle” package to perform pseudotime analysis [33,34,35]. We employed the ‘estimateSizeFactors’ and ‘estimateDispersions’ functions to normalize the data and compute dispersion values. Unsupervised ordering genes were identified based on mean expression and dispersion empirical thresholds according to standard Monocle 2 pipelines. Dimensionality reduction was performed using the ‘DDRTree’ algorithm via ‘reduceDimension’. To define trajectory directionality in the ‘orderCells’ function, root states were designated based on clear biological reference criteria: for the monocyte/macrophage lineage, Cluster 4—which uniquely exhibited high expression of the undifferentiated monocyte marker *Ear2* and low mature macrophage marker expression (*Adgre1*)—was assigned as the biological root state. For dermal fibroblasts, the computational root state was assigned to the baseline stromal state based on its retention of canonical homeostatic fibroblast markers, providing a reference baseline to project the putative transcriptional continuum toward specialized hormone-receptor-associated states. Cells were projected using ‘plot_cell_trajectory’.

### 2.11. Primary Dermal Fibroblast Culture, Hormone Stimulation, and Enzyme-Linked Immunosorbent Assay (ELISA)

Primary mouse dermal fibroblasts were isolated from the dorsal skin of age-matched female BALB/c mice as described previously and followed the protocol of the previous study [36]. Primary dermal fibroblasts (passages ≤ 3) were resuspended in complete medium and evaluated for viability via 0.4% trypan blue exclusion, ensuring viability exceeded 90%. Cells were seeded into 24-well flat-bottom culture plates at a density of 1 × 10^6^ cells/well in 1 mL complete medium and cultured overnight to allow adherence. After washing twice with prewarmed DPBS (D485558, Aladin, Shanghai, China), the cells were starved in 1 mL DMEM (11965092, Gibco, Carlsbad, CA, USA) supplemented with 0.5% fetal bovine serum (FBS) (A5256701,Gibco, Carlsbad, CA, USA) for 12 h to minimize serum interference. Fibroblasts were then stimulated with 17β-estradiol (100 pg/mL) (B24215, MedMol, Shanghai, China), progesterone (100 ng/mL) (B20572, MedMol, Shanghai, China), or recombinant mouse growth hormone (50 ng/mL) (HY-P70261, MedChemExpress, Shanghai, China) according to the previous studies [37,38], while the control group remained untreated. Cell culture supernatants were harvested at 48 h post-stimulation for subsequent analysis. Progranulin (PGRN), Gas6, and TGF-β2 of cell culture supernatants were determined using commercial mouse ELISA kits according to the manufacturer’s instructions (EK2184EG, EK2384, EK9162, Multi Sciences, Hangzhou, China).

### 2.12. Multiplex Immunofluorescence (mIF) Staining

Dorsal skin paraffin sections obtained as described for H&E staining were deparaffinized in xylene, rehydrated through graded ethanol series, and subjected to heat-induced antigen retrieval. mIF staining was conducted using a multiplex fluorescent immunohistochemistry kit (G1256, Servicebio, Wuhan, China) according to the manufacturer’s instructions. Tissue sections were sequentially incubated with primary antibodies against F4/80 (81668-1-RR, Proteintech, Wuhan, China) and MerTK (98798-1-RR, Proteintech, Wuhan, China), followed by corresponding secondary horseradish peroxidase-conjugated antibodies and tyramide signal amplification fluorophores. Nuclei were counterstained with DAPI. Multispectral fluorescent images were acquired using a confocal laser scanning microscope. The proportion of MerTK^+^ cells among F4/80^+^ cells was quantified across five randomly selected high-power fields (400×) per section using QuPath (v 0.5.1) by two blinded assessors.

### 2.13. Statistical Analysis

Data are presented as mean ± standard error of the mean (SEM), with all numerical values of phenotypic experiments rounded to two decimal places. Statistical analyses were performed using R (v 4.3.3), employing Student’s *t*-test, or one-way ANOVA followed by Tukey’s post hoc multiple comparison test. Statistical significance thresholds were set as *p* < 0.05 (* *p* < 0.05, ** *p* < 0.01, *** *p* < 0.001). The cell experiments were repeated 3 times. Other experiments were performed using a single, age-matched cohort (n = 6/group for phenotypic experiments, n = 3/group for sequencing).

## 3. Results

### 3.1. Pregnancy Improved Psoriasis-like Dermatitis Induced by IMQ in Mice

Female mice were treated with IMQ for 5 consecutive days and euthanized on day 6. Mice were randomly assigned to three groups: a vehicle-treated group (control group), an IMQ-induced group (IMQ group), and an IMQ-induced group during pregnancy (IMQ + Pregnancy group).

**Figure 1:**
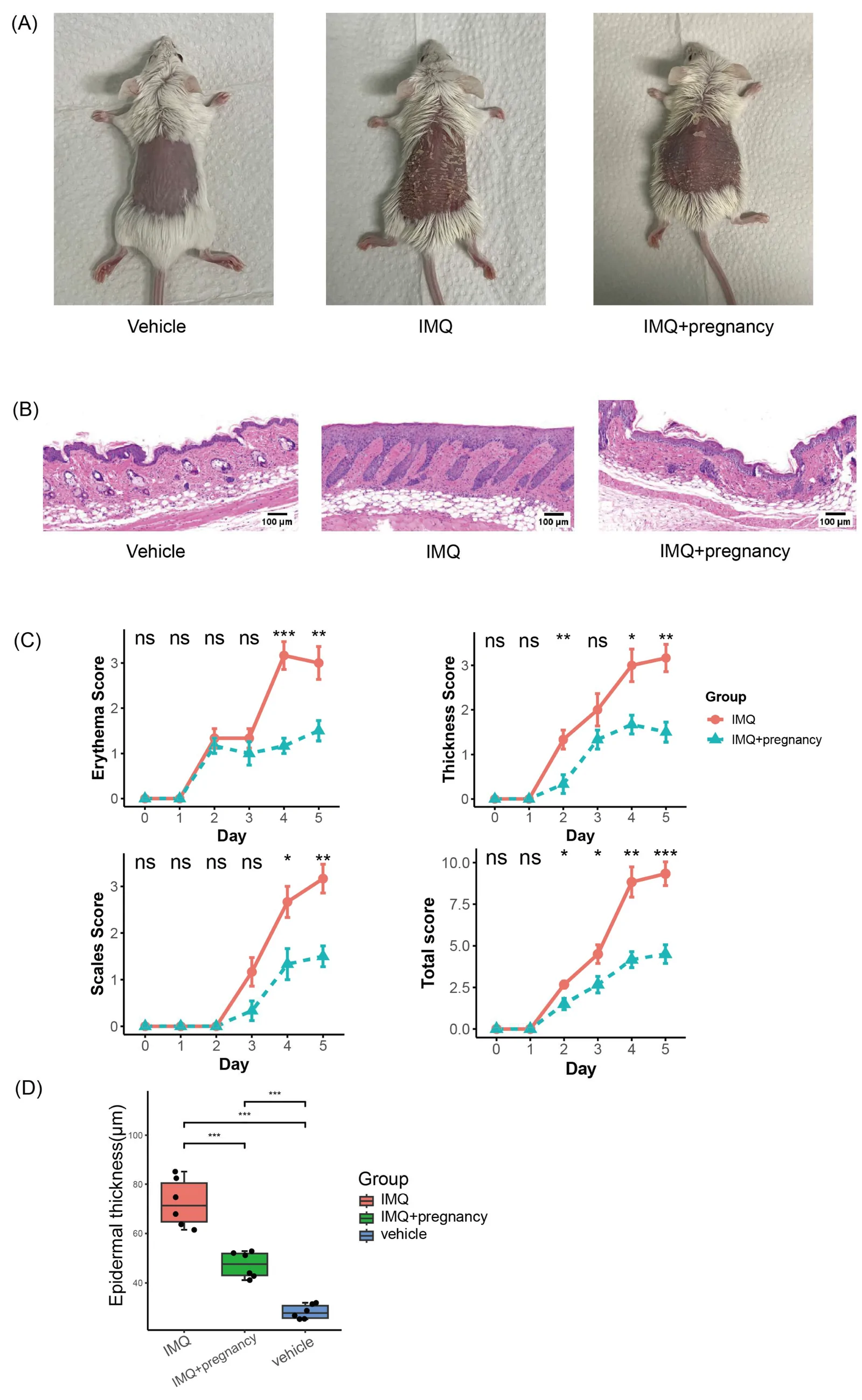
(**A**) Representative clinical photos of mice dorsal skin tissues on the 6th day treated with IMQ. (**B**) H&E staining of mice dorsal skin tissues in each group. Bar = 100 μm. (**C**) mPASI scores assessing erythema, scaling, thickness and cumulative disease severity. (**D**) Quantification of epidermal thickness. Data were presented as mean ± SEM. n = 6 per experiment. * *p* < 0.05, ** *p* < 0.01, *** *p* < 0.001.

As expected, compared with the vehicle control group, IMQ treatment significantly induced psoriasis-like lesions (Figure 1A). Histopathological analysis further confirmed characteristic psoriasis-related pathological features in IMQ-treated mice, including epidermal thickening, hyperkeratosis, epidermal hyperplasia, acanthosis, and inflammatory cell infiltration (Figure 1B). Quantification of epidermal thickness (Figure 1D) confirmed a significant reduction in the IMQ + pregnancy group compared with the IMQ group (47.36 ± 2.15 μm vs. 72.53 ± 4.01 μm; Tukey’s post hoc test, *p* < 0.0001).

mPASI scores were quantified to evaluate disease progression and severity (Figure 1C). Specifically, at day 5, the IMQ + pregnancy group exhibited a significant reduction across all parameters compared with the IMQ group. The erythema score was significantly attenuated (1.43 ± 0.31 vs. 3.00 ± 0.37, Student’s *t*-test, *p* = 0.0076), as was the scales score (1.54 ± 0.25 vs. 3.17 ± 0.31, Student’s *t*-test, *p* = 0.0017), and the thickness score (1.52 ± 0.22 vs. 3.17 ± 0.31, Student’s *t*-test, *p* = 0.0017). Consequently, the total mPASI score of IMQ + pregnancy group was significantly lower than that of IMQ group (4.50 ± 0.56 vs. 9.33 ± 0.71, Student’s *t*-test, *p* < 0.0001).

### 3.2. Bulk RNA-Seq Unveiled Complex Transcriptomic Profiles Associated with Pregnancy in the IMQ-Induced Psoriatic Dermatitis Model

**Figure 2:**
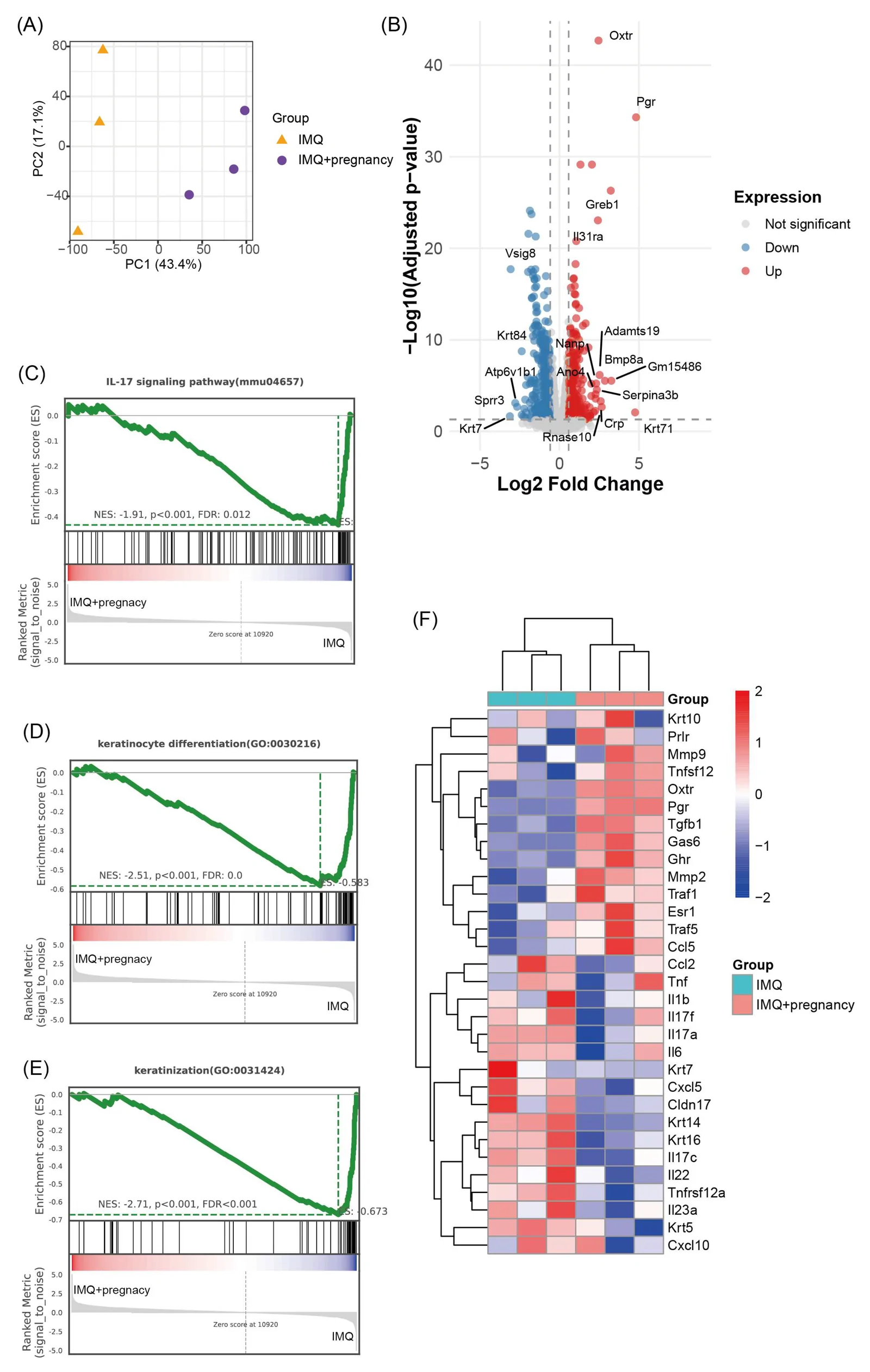
(**A**) PCA of skin transcriptional profiles. (**B**) Volcano plots exhibit the up- and downregulated genes in each dataset. Blue dots represent downregulated DEGs and red dots represent upregulated DEGs of IMQ + pregnancy group. The *p*-values shown are adjusted for multiple comparisons using the FDR method. Enrichment plot of genes involved in IL-17 signaling pathway (**C**), keratinocyte differentiation (**D**) and keratinization (**E**) conducted by GSEA. (**F**) The heatmap shows the expression levels of key genes between the IMQ group and IMQ + pregnancy group.

To further characterize transcriptomic differences in lesional skin tissues between the IMQ and IMQ + Pregnancy groups, we performed bulk RNA-seq analysis. PCA revealed distinct separation of transcriptional profiles between the two groups along principal components (Figure 2A). DEGs were visualized in a volcano plot, with key genes labeled—including hormone-associated genes (e.g., *Oxtr*, *Pgr*, *Greb1*) (Figure 2B) [39].

GSEA demonstrated downregulation of the IL-17 signaling pathway, keratinocyte differentiation, and keratinization in the IMQ + Pregnancy group (Figure 2C–E), which correlated with attenuated inflammatory responses and reduced epidermal thickening [7]. Additionally, we performed KEGG and GO enrichment analyses on upregulated and downregulated DEGs separately. KEGG analysis revealed downregulation of the IL-17 and TNF signaling pathways, alongside upregulation of Th17 cell differentiation, Th1/Th2 cell differentiation, and the PD-L1 expression/PD-1 checkpoint pathway in cancer (Figure S1A,B). GO analysis showed downregulation of epidermal development, skin development, and keratinocyte differentiation, as well as upregulation of T-cell activation regulation, leukocyte proliferation, and mononuclear cell proliferation (Figure S2A,B). Collectively, these enrichment results revealed complex alterations in the cutaneous immune microenvironment and keratinocyte proliferation.

To further delineate group-specific differences, we used a heatmap to visualize the expression levels of key genes (Figure 2F). Genes encoding key pro-inflammatory factors (e.g., *Il17a*, *Il17f*, *Il17c*, *Tnf*, *Il1b*, *Il6*, *Il22*, *Il23a*) [7] and those associated with keratinization (e.g., *Krt14*, *Krt16*, *Krt5*) [7] were downregulated in the IMQ + Pregnancy group. In contrast, hormone-associated genes (e.g., *Prlr*, *Oxtr*, *Pgr*, *Ghr*, *Esr1*) [39,40] and genes encoding anti-inflammatory factors (e.g., *Tgfb1*, *Gas6*) [41,42] were upregulated.

Collectively, bulk RNA-seq analysis delineated the complex transcriptional changes underlying pregnancy-associated modulation of the IMQ-induced psoriatic dermatitis model.

### 3.3. Single-Cell RNA-Seq Analysis Revealed Transcriptomic Variations Associated with Pregnancy in Skin Cellular Composition and Predicted Ligand–Receptor Communication in the IMQ-Induced Psoriatic Model

**Figure 3:**
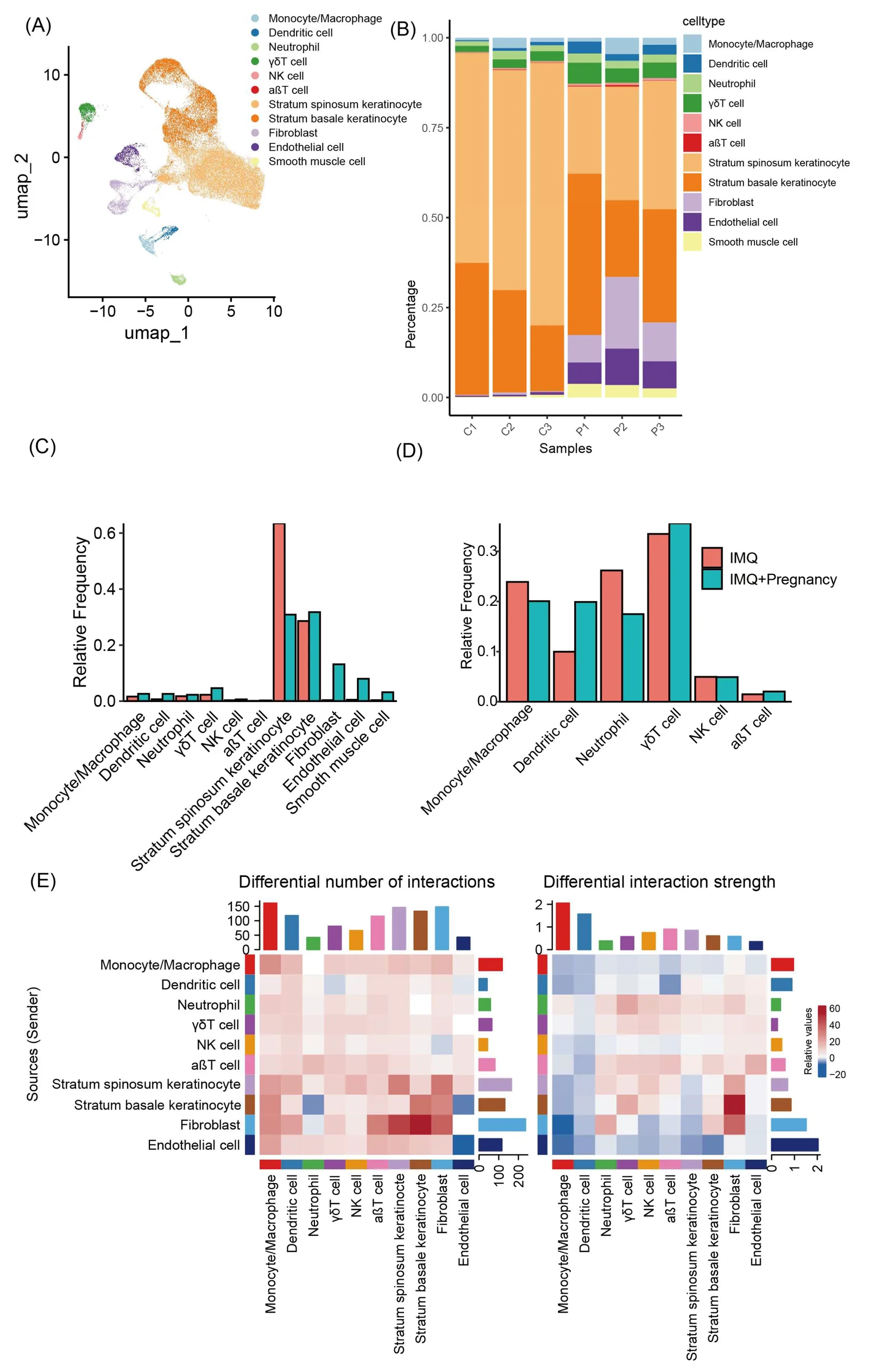
(**A**) UMAP visualization of cells from lesional skin; distinct cell types are represented by different colors. (**B**) Proportions of distinct cell types in each sample. Samples C1, C2, and C3 were derived from the IMQ group, while P1, P2, and P3 were from the IMQ + Pregnancy group. (**C**) Relative frequency of distinct cell types in the IMQ group versus the IMQ + Pregnancy group. (**D**) Relative frequency of distinct immune cell subsets in the IMQ group versus the IMQ + Pregnancy group. (**E**) Predicted differential number of intercellular interactions and predicted differential intercellular communication strength between the IMQ group and IMQ + Pregnancy group. Red indicates upregulation in the IMQ + Pregnancy group, while blue indicates downregulation.

Given the complex transcriptional findings from bulk RNA-seq, we systematically investigated the cellular composition and functional states of distinct cell types by performing scRNA-seq on total skin cells isolated from lesional skin tissues of the IMQ group (n = 3) and IMQ + Pregnancy group (n = 3). Following quality control filtering, we generated a total of 57,056 high-quality single-cell transcriptomes. Subsequent selection of variable genes and UMAP dimensionality reduction enabled the identification of 11 distinct cell types based on lineage-specific markers, including smooth muscle cells, endothelial cells, fibroblasts, stratum basale keratinocytes, stratum spinosum keratinocytes, αβT cells, natural killer (NK) cells, γδT cells, neutrophils, dendritic cells, and monocytes/macrophages (Figure 3A and Figure S3A).

The IMQ + Pregnancy group exhibited a reduction in the proportion of stratum spinosum keratinocytes (Figure 3A,B). Among immune cell populations, we observed a decrease in the proportions of monocytes/macrophages and neutrophils, alongside an increase in dendritic cells (Figure 3C,D). These proportional shifts suggest that these cell populations correlate with the pregnancy-associated transcriptomic shifts observed in the model.

We further employed the R package CellChat to analyze potential changes in intercellular communication between the two groups. Overall, the IMQ + Pregnancy group showed a greater predicted number of cell–cell interactions but reduced predicted interaction strength compared with the IMQ group (Figure S3B). Notably, fibroblasts, monocytes/macrophages, and keratinocytes displayed both a higher number and greater strength of predicted interactions than other cell types (Figure 3E), reflecting high predicted computational interaction potential among these populations within the lesional microenvironment.

Additionally, comparison of global intercellular information flow revealed increased predicted information flow of the THBS, IL-2, VISTA, GRN, Gas6, TGF-β, FGF, and CD200 pathways, as well as decreased predicted information flow of the TNF, IL-17, MIF, and IFN-II pathways in the IMQ + Pregnancy group (Figure S4). These changes indicate attenuated inflammatory responses and highlight potential candidate pathways associated with pregnancy-related variations in psoriatic pathogenesis.

### 3.4. Hormone-Receptor-Associated Fibroblast States Correlate with Pregnancy-Associated Shifts in the Psoriatic Dermatitis Model

Hormones (e.g., estrogen, progesterone, growth hormone, prolactin) exert profound regulatory effects on pregnancy. Additionally, bulk RNA-seq analysis showed that hormone-associated genes were significantly upregulated in the IMQ + Pregnancy group. To further clarify the expression patterns of these hormone-related genes, we analyzed scRNA-seq data, which revealed that key hormone receptor genes (e.g., *Esr1*, *Pgr*, *Ghr*, *Prlr*) exhibited higher expression levels in fibroblasts and keratinocytes (Figure S5A), which might indicate enhanced hormonal responsiveness of these cells.

**Figure 4:**
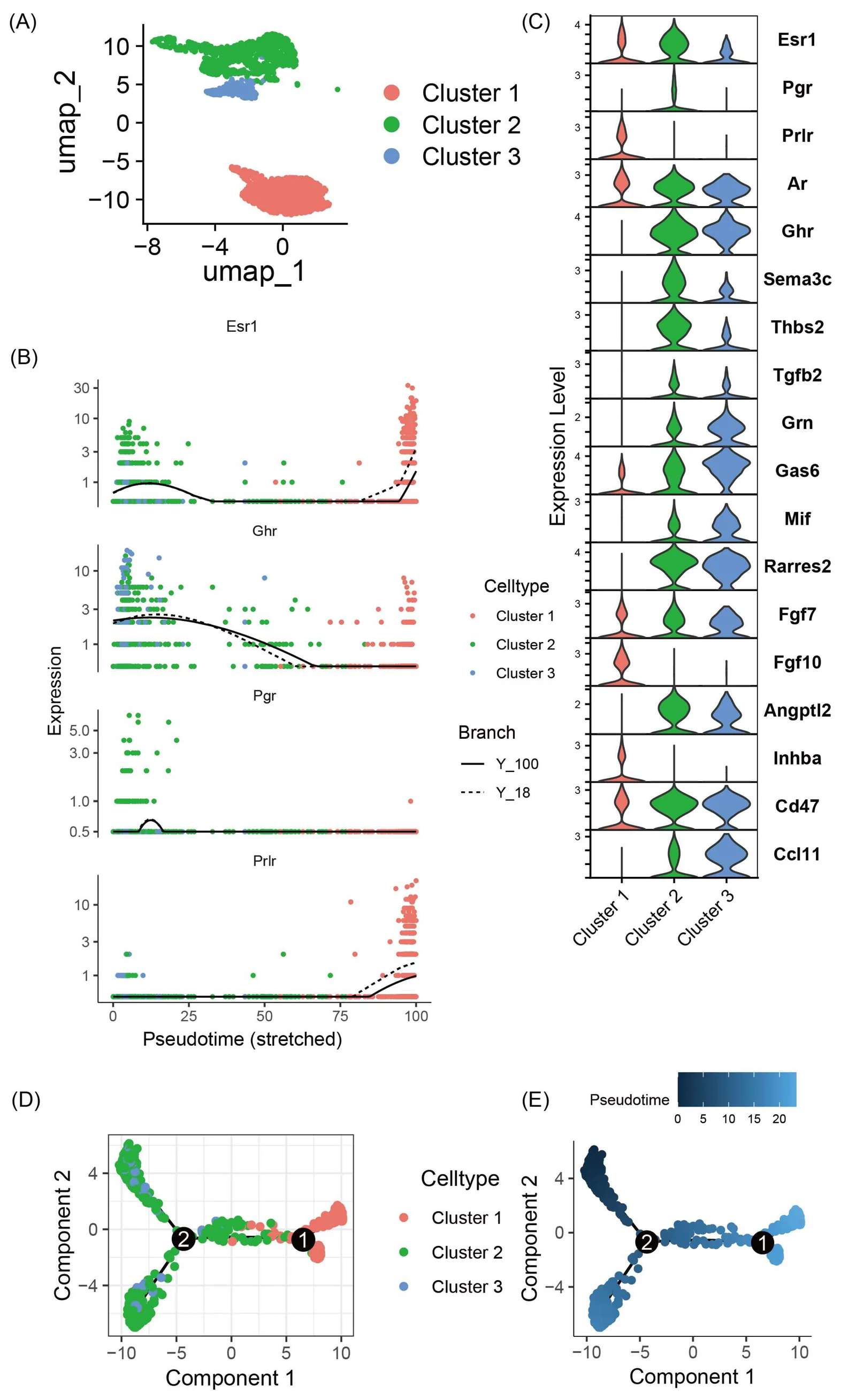
(**A**) UMAP visualization of fibroblast subclusters; distinct subclusters are represented by different colors. (**B**) Hormone-related gene expression dynamics over pseudotime. (**C**) Violin plots of specific genes in fibroblast subclusters. Monocle-inferred pseudotime trajectory of fibroblasts, colored by (**D**) subcluster identity and (**E**) pseudotime.

To further characterize the pregnancy-associated transcriptomic shifts in fibroblasts, we subdivided fibroblasts into three distinct clusters: *Esr1*^low^ *Prlr*^+^ fibroblasts (Cluster 1), *Esr1*^high^ *Pgr*^+^ fibroblasts (Cluster 2) and *Esr1*^low^ *Ghr*^+^ fibroblasts (Cluster 3) (Figure 4A and Figure S5C). Clusters 2 and 3 showed high expression of anti-inflammatory transcripts (e.g., *Gas6*, *Sema3c*, *Tgfb2*, *Grn*, *Angptl2*)—encoding molecules known to promote macrophage polarization toward the M2 phenotype [43,44,45]. However, Clusters 2 and 3 also highly expressed genes linked to keratinocyte proliferation and inflammation (e.g., *Mif*, *Rarres2*, *Fgf7*) (Figure 4C) [46,47]. In contrast, Cluster 1 primarily expressed genes involved in promoting keratinocyte proliferation (e.g., *Fgf7*, *Fgf10*) (Figure 4C).

Pseudotime inference of fibroblast expression profiles suggested a potential transcriptional continuum spanning from Clusters 2/3 to Cluster 1 (Figure 4D,E). Rather than demonstrating physical cell state transition over time, this computational continuum reflected a spectrum of distinct hormone-receptor-associated transcriptional states. Dynamic changes in the expression levels of key hormone-related genes across this continuum were observed (Figure 4B).

GO enrichment analysis of fibroblast DEGs between the two groups demonstrated that fibroblasts in the IMQ + Pregnancy group upregulated terms related to cellular response to peptide hormone stimulus and response to steroid hormones while downregulating terms such as keratinization and defense response to bacterium (Figure S5B). Comparison of global intercellular information flow showed increased predicted information flow of anti-inflammatory pathways (e.g., GRN, Gas6, ANGPTL) and keratinocyte-proliferation pathways (e.g., FGF), alongside decreased predicted information flow of pro-inflammatory pathways (e.g., MIF) (Figure S6).

Collectively, these findings indicate that fibroblasts exhibit transcriptional heterogeneity during pregnancy-associated modulation. Rather than operating purely as anti-inflammatory cells, they display a complex, dual transcriptional phenotype—simultaneously expressing transcripts for immunoregulatory factors alongside inflammatory mediators involved in keratinocyte modulation and leukocyte recruitment.

### 3.5. Pregnancy-Associated Transcriptomic Profiles Exhibit Downregulation of Genes Linked to Pathological Epidermal Proliferation and an Anti-Inflammatory Keratinocyte Phenotype

**Figure 5:**
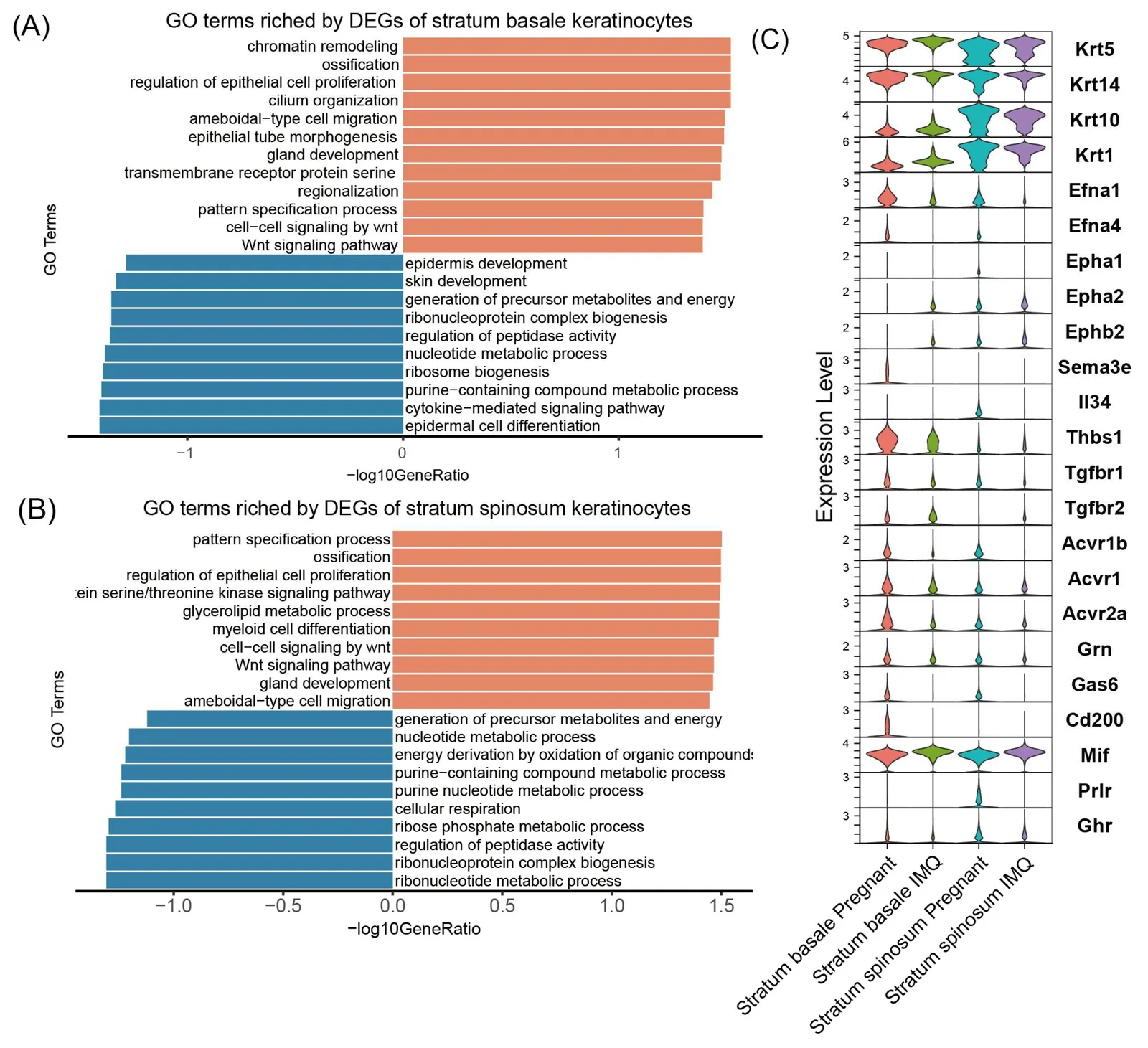
The top 20 terms from GO enrichment analysis of upregulated and downregulated DEGs of stratum basale keratinocytes (**A**) and stratum spinosum keratinocytes (**B**), sorted by GeneRatio. (**C**) Violin plots of specific genes in keratinocytes; “stratum basale pregnancy” and “stratum spinosum pregnancy” refer to stratum basale keratinocytes and stratum spinosum keratinocytes isolated from the IMQ + pregnancy group, respectively; conversely, “stratum basale IMQ” and “stratum spinosum IMQ” denote stratum basale keratinocytes and stratum spinosum keratinocytes derived from the IMQ group.

Pathological keratinocyte proliferation is a hallmark of psoriasis. GO enrichment analysis of DEGs revealed remission of excessive keratinocyte proliferation, metabolic hyperactivity, and synthetic hyperactivity, alongside skin barrier repair and a shift toward an anti-inflammatory state (Figure 5A,B).

Violin plots show the expression levels of specific genes in keratinocytes (Figure 5C). Keratinocytes in the IMQ + Pregnancy group exhibited high expression of *Efna1*—a gene critical for normal keratinization and keratinocyte differentiation [48]. Genes encoding anti-inflammatory factors (e.g., *Grn*, *Gas6*) were upregulated in this group, and genes encoding receptors of the TGF-β superfamily (e.g., *Tgfbr1*, *Tgfbr2*, *Acvr1b*) were also upregulated. Conversely, *Mif*, the gene encoding the pro-inflammatory factor, was downregulated.

Notably, stratum basale keratinocytes showed high expression of *Sema3e*—a molecule known to suppress Th1-type immune responses and reduce neutrophil accumulation [49]. Additionally, *Il34* (a cytokine reported to be downregulated in psoriatic lesional skin and critical for keratinocyte differentiation) was upregulated in stratum spinosum keratinocytes of the IMQ + Pregnancy group [50]. Consistent with these findings, the predicted information flow of EPHA, TGF-β, Gas6, and GRN pathways was enriched in the IMQ + Pregnancy group (Figures S10 and S11).

### 3.6. Monocytes/Macrophages Display M2-like Transcriptional Signatures in the Pregnant Group

**Figure 6:**
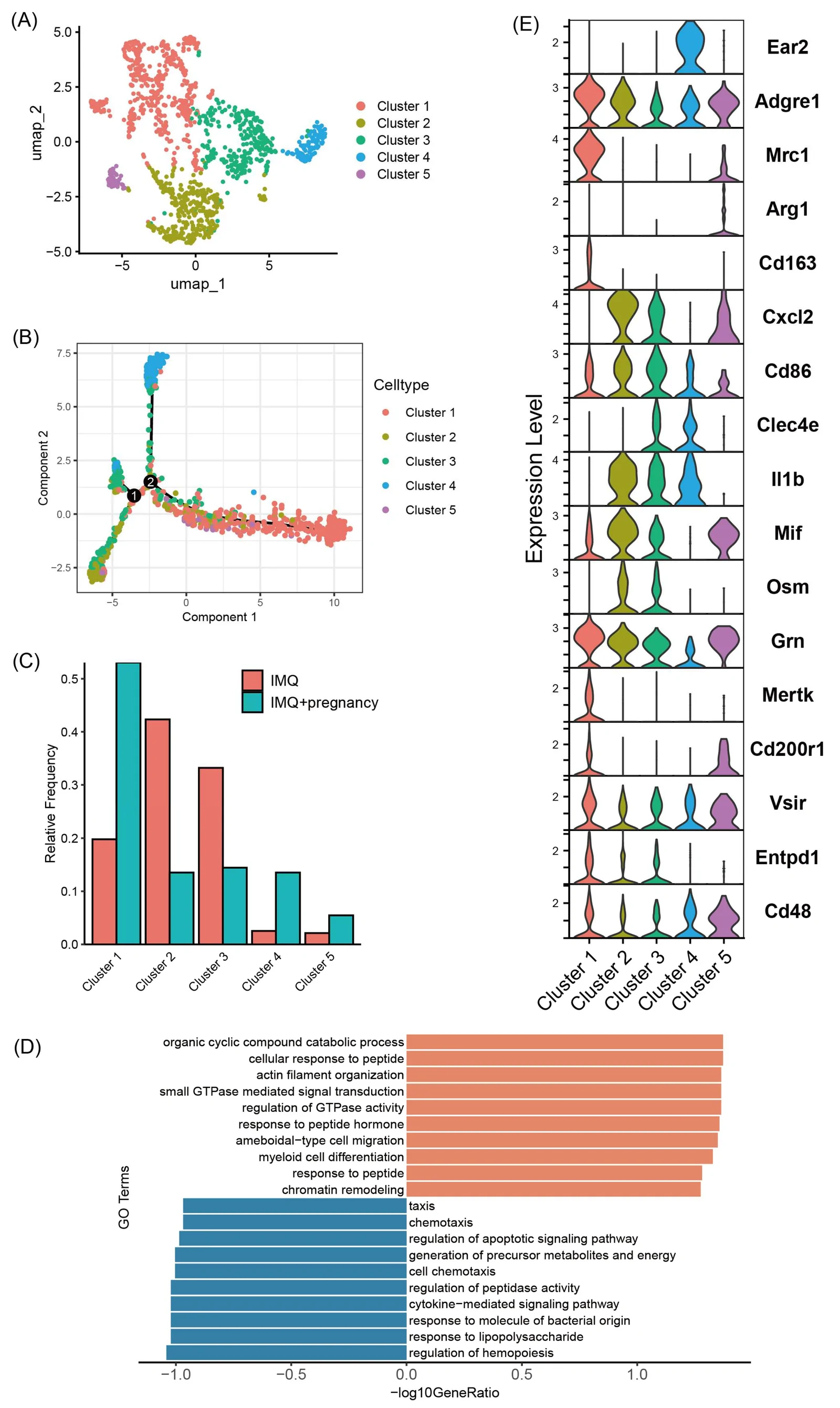
(**A**) UMAP visualization of monocyte/macrophage subclusters; distinct subclusters are represented by different colors. (**B**) Monocle-inferred pseudotime trajectory of monocytes/macrophages colored by subcluster identity. (**C**) Relative frequency of monocyte/macrophage subclusters in the IMQ group versus the IMQ + Pregnancy group. (**D**) The top 20 terms from GO enrichment analysis of upregulated and downregulated DEGs of monocytes/macrophages, sorted by GeneRatio. (**E**) Violin plots of signature or specific genes in monocyte/macrophage subclusters.

Cell–cell interaction analysis emphasized the potential critical association of the Gas6, GRN and TGF-β pathways with pregnancy-associated transcriptomic shifts in the IMQ-induced psoriatic model. Monocytes/macrophages might represent key cell types in these predicted pathways and engage in intercellular crosstalk with fibroblasts and keratinocytes (Figures S7 and S8). To further characterize this role, we focused on the monocyte/macrophage population and identified five distinct subclusters (Figure 6A).

Among these subclusters, Cluster 1, 4, and 5 exhibited an increase in proportion in the IMQ + Pregnancy group (Figure 6C), suggesting their potential transcriptomic association with attenuated psoriatic inflammation. Violin plots were used to visualize the expression levels of signature genes in each subcluster (Figure 6E): Cluster 1 exhibited high expression of *Adgre1*, *Mrc1*, and *Cd163*—genes associated with M2-like macrophage polarization—indicating it possesses M2-like transcriptional signatures; Cluster 5 similarly expressed high levels of *Adgre1*, *Mrc1*, and *Arg1*, suggesting it also represents M2-like transcriptional signatures; Cluster 4 showed high expression of *Ear2* and low expression of *Adgre1*, suggesting it may represent monocyte-like or M0-like transcriptional signatures; Clusters 2 and 3 expressed high levels of *Adgre1* and *Cd86* (markers typically linked to pro-inflammatory macrophage states), alongside genes encoding pro-inflammatory factors (e.g., *Il1b*, *Mif*, *Osm*), indicating that these two subclusters possess M1-like transcriptional signatures [51].

Notably, Clusters 1 and 5 expressed high levels of genes encoding anti-inflammatory factors (e.g., *Grn*) and co-inhibitory immune checkpoint molecules (e.g., *Vsir*, *Cd200r1*). Cluster 1 further showed elevated expression of *Mertk* and *Entpd1*: *Mertk* encodes a receptor for Gas6, which highlights potential intercellular crosstalk among monocytes/macrophages, fibroblasts, and keratinocytes [41]; *Entpd1* encodes CD39, a molecule known to suppress the differentiation of Th1/Th17 pro-inflammatory T cells [52].

Pseudotime trajectory analysis revealed Cluster 4 localized at the starting position of the trajectory, with Cluster 1/5 distributed on the right and Cluster 2/3 on the left (Figure 6B), which indicated their distinct transcriptional signatures.

GO enrichment analysis of monocyte/macrophage DEGs showed that pregnancy upregulated terms related to hormone response and anti-inflammatory differentiation, while downregulating terms associated with pro-inflammatory activation and pathological migration (Figure 6D)—consistent with the phenotypic shift observed in the subclusters.

### 3.7. In Vitro Fibroblast Secretion Assessment and In Situ Macrophage MerTK Evaluation

To test whether the computationally predicted pathways reflect measurable protein changes, we evaluated primary mouse dermal fibroblasts treated with 17β-estradiol, progesterone or growth hormone for 48 h. ELISA quantification revealed that individual stimulation with estradiol, progesterone, or growth hormone significantly elevated extracellular concentrations of PGRN in culture supernatants compared with the untreated control at 48 h post-stimulation ([591.80 ± 30.02 pg/mL] in the control group vs. [890.48 ± 56.36 pg/mL, Tukey’s post hoc test, *p* = 0.0019] with estradiol, [890.83 ± 7.10 pg/mL, Tukey’s post hoc test, *p* = 0.0018] with progesterone, and [993.96 ± 87.16 pg/mL, Tukey’s post hoc test, *p* = 0.0034] with growth hormone; Figure S12A). For Gas6 secretion, extracellular levels rose significantly from 293.06 ± 24.04 pg/mL in controls to 521.36 ± 20.82 pg/mL (Tukey’s post hoc test, *p* = 0.0028) following estradiol challenge and 484.18 ± 27.35 pg/mL (Tukey’s post hoc test, *p* = 0.0081) with growth hormone exposure (Figure S12B). In contrast, progesterone stimulation led to an increase in Gas6 that exhibited borderline statistical significance (422.23 ± 42.18 pg/mL, Tukey’s post hoc test, *p* = 0.060; Figure S12B). Under identical in vitro culture conditions, TGF-β2 protein remained below the lower limit of detection in both control and hormone-stimulated supernatants.

We further examined lesional skin tissues by multiplex immunofluorescence to assess the Gas6 receptor MerTK on macrophages in vivo. MerTK signals were primarily localized to F4/80^+^ cells (Figures S13 and S14). Notably, the proportion of MerTK^+^ cells among F4/80^+^ cells was significantly higher in the IMQ + Pregnancy group than in the non-pregnant IMQ group (0.76 ± 0.043 vs. 0.33 ± 0.056, Tukey’s post hoc test, *p* < 0.001; Figure S15). These results demonstrate that gestational hormones can directly enhance fibroblast secretion of PGRN and Gas6 in vitro, and that the pregnancy status correlates with increased macrophage MerTK expression in psoriatic lesions in vivo.

## 4. Discussion

Psoriasis is a chronic inflammatory skin disorder that severely damages the quality of life and imposes a considerable burden [1]. In women, hormonal fluctuations—including pregnancy, which often ameliorates psoriasis—exert profound effects on disease manifestation [4], but the underlying mechanisms of this protection remain poorly understood. To address this gap, we used the IMQ-induced psoriatic model to recapitulate pregnancy-related changes in patients and integrated bulk and single-cell RNA-seq analysis to characterize cutaneous microenvironmental shifts. Previous studies demonstrated that estrogen and progesterone can reduce the severity of psoriasis by affecting immune cells directly [2,12]. Consistent with these established immune effects, our data confirmed downregulation of *Il1b*, *Il17a/f*, and *Tnf* effector transcripts, alongside a reduced frequency of neutrophils and a shift of monocytes/macrophages toward M2-like transcriptional signatures. Extending beyond this immune-centered view, our study demonstrated that dermal fibroblasts express diverse hormone receptor transcripts and may participate in coordinate immunoregulatory transcriptomic networks with monocytes/macrophages. Also, our study unraveled transcriptomic functional alterations in keratinocytes—important participants in psoriasis pathogenesis.

Bulk RNA-seq analysis revealed alleviation of Th1/Th17-type inflammation and significant upregulation of hormone-associated genes (e.g., *Pgr*, *Oxtr*). Notably, further investigation showed high expression of these hormone-associated genes in fibroblasts. Via cell–cell interaction analysis, we detected elevated expression of genes encoding anti-inflammatory factors—including *Tgfb2*, *Gas6*, and *Grn*—in *Esr1*^high^ *Pgr*^+^ fibroblasts and *Esr1*^low^ *Ghr*^+^ fibroblasts.

Notably, despite the suppression of downstream IL-17 inflammatory effector transcripts (e.g., *Il17a*, *Il17f*, *Il1b*, and *Tnf*) during pregnancy, KEGG pathway analysis revealed an enrichment of Th17 cell differentiation and Th1/Th2 cell differentiation. This difference can be explained by our single-cell data on the myeloid microenvironment. In the pregnant group, monocytes and macrophages shifted toward M2-like transcriptional signatures. These transcriptomic profiles correlate with pathways involved in Th2 cell differentiation and exhibit lower activation scores of Th1 and Th17 cells. Therefore, the high enrichment scores of these differentiation pathways likely reflect upstream cellular changes and feedback mechanisms within the pregnant skin microenvironment, rather than actual proliferation of Th1 and Th17 cells.

In cutaneous biology, TGF-β exerts complex functions: while it synergizes with IL-6 to promote Th17 differentiation in acute immune responses [53], it also functions to restrain excessive keratinocyte proliferation and facilitate anti-inflammatory macrophage differentiation [54,55,56]. Our single-cell RNA sequencing and CellChat analysis computationally inferred enriched Tgfb2 transcripts in dermal fibroblasts and predicted TGF-β communication toward myeloid cells. However, when primary dermal fibroblasts were stimulated in vitro with 17β-estradiol, progesterone, or growth hormone, TGF-β2 protein remained below the lower limit of detection in culture supernatants across both untreated and hormone-stimulated conditions. This discrepancy underscores the critical necessity of experimentally evaluating computational ligand–receptor predictions. In native tissue microenvironments, TGF-β family members are frequently sequestered as latent complexes within the extracellular matrix and necessitate specific mechanical tension or integrin-mediated enzymatic cleavage for active release, which may be absent in isolated fibroblast cultures. Alternatively, non-stromal populations within the cutaneous infiltrate, such as regulatory T cells or specific myeloid subsets, might serve as the predominant physiological source of functional TGF-β in vivo [54,55]. Consequently, while *Tgfb2* transcription is detected in the stroma, our experimental observations do not support stromal TGF-β2 as a directly secreted mediator in response to gestational hormones in vitro.

Gas6 acts as a primary ligand for the TAM receptor tyrosine kinase family (Tyro3, Axl, and MerTK) [41]. Engagement of Gas6 with MerTK promotes the clearance of apoptotic debris and actively skews macrophages toward an M2-like homeostatic phenotype while suppressing pro-inflammatory cascades such as NF-κB and downstream IL-1β release [57]. Notably, prior clinical and preclinical investigations have documented diminished expression of Gas6 and TAM receptors within active psoriatic plaques [58]. Consistent with our computational predictions linking stromal Gas6 to myeloid MerTK, our in vitro assays demonstrated that primary dermal fibroblasts directly enhance Gas6 secretion upon stimulation with gestational hormones. Specifically, 48 h challenge with 17β-estradiol or growth hormone triggered significant elevations in extracellular Gas6 protein, whereas progesterone exposure resulted in an upward trend that reached borderline statistical significance. Furthermore, in situ mIF verified that the Gas6 receptor MerTK was expressed on F4/80^+^ macrophages in lesional skin and was significantly elevated in a positive cell ratio in pregnant mice. Together, these parallel in vitro experiments and in vivo tissue findings provide protein-level evidence supporting a potential intercellular crosstalk between fibroblasts and macrophages through the Gas6-MerTK axis that may participate in pregnancy-associated disease modulation.

Grn encodes progranulin (PGRN), an autocrine and paracrine glycoprotein that exhibits potent immunoregulatory properties across cutaneous and mucosal tissues [59]. Mechanistically, PGRN can competitively bind to TNF receptors (TNFR1 and TNFR2), thereby antagonizing TNF-α-mediated pro-inflammatory cascades, dampening neutrophil and macrophage activation, and promoting M2-like macrophage differentiation [60]. In preclinical models of psoriasis, recombinant PGRN administration has been demonstrated to substantially alleviate IMQ-induced epidermal hyperplasia and inflammatory infiltration [61]. In line with these reports, our single-cell data identified robust *Grn* expression within specific fibroblast clusters. Experimentally, our in vitro experiments confirmed that 17β-estradiol, progesterone, and growth hormone each individually and significantly enhanced extracellular PGRN secretion from primary dermal fibroblasts at 48 h post-stimulation. These results demonstrate that multiple gestational endocrine factors directly augment fibroblast-derived PGRN release, identifying dermal fibroblasts as an active stromal source of this anti-inflammatory factor that may cooperate with Gas6 to modulate the cutaneous myeloid microenvironment.

Notably, we also observed high expression of FGFs (e.g., *Fgf7*, *Fgf10*) across all fibroblast subclusters. Consistent with prior reports, FGF7 and FGF10 have been shown to be upregulated in psoriatic lesional skin [62]. FGF7 promotes keratinocyte proliferation and induces keratinocytes to upregulate TNF-α expression, thereby exacerbating psoriatic pathology [63]. Additionally, treatment with an FGF10 monoclonal antibody has been demonstrated to attenuate psoriasis-like lesions in guinea pigs [64]. *Esr1*^low^ *Ghr*^+^ fibroblasts and *Esr1*^high^ *Pgr*^+^ fibroblasts express genes encoding both FGF and anti-inflammatory factors (e.g., *Gas6*, *Grn*), indicating a complex role of fibroblasts in psoriasis during pregnancy. The complex interplay between estrogen, progesterone, and growth hormone may correlate with shifting transcriptomic states of fibroblasts, offering candidate pathways for future functional validation. *Esr1*^low^ *Prlr*^+^ fibroblasts specifically exhibit high expression levels of *Fgf7* and *Fgf10*. Following delivery, sustained elevation of serum prolactin may stimulate fibroblast-derived FGF secretion, potentially leading to the exacerbation of psoriasis.

An essential mechanistic question is whether the observed fibroblast alterations represent a primary direct response to gestational hormones, a secondary consequence of alleviated local inflammation, or a parallel coordinated process. Because dermal fibroblasts express canonical hormone receptors (*Esr1*, *Pgr*, *Ghr*, *Prlr*) and primary fibroblasts directly upregulated Gas6 and PGRN secretion upon gestational hormone stimulation in vitro, fibroblasts possess the intrinsic capacity to directly sense endocrine cues. However, given that direct hormonal inhibition of myeloid cells also rapidly dampens local cytokine secretion [12], stromal changes in vivo may additionally represent secondary adaptations to a resolving inflammatory milieu, or operate in parallel as a coordinate facet of systemic pregnancy-associated modulation. Lineage-specific receptor conditional knockout models will be required to definitively distinguish between these possibilities.

Pregnancy markedly downregulated the expression of genes associated with pathological proliferation and pro-inflammatory phenotype of keratinocytes. Specifically, keratinocytes exhibited elevated expression of genes encoding anti-inflammatory factors (e.g., *Grn*, *Gas6*) and downregulated the expression of *Mif*. These findings underscore the critical role of keratinocytes in shaping the anti-inflammatory microenvironment during pregnancy. Furthermore, the expression of *Ghr* and *Prlr* in keratinocytes suggests that beyond the regulatory effects of fibroblasts and immune cells, hormones may associate with keratinocyte proliferation and anti-inflammatory transcriptomic profiles—providing additional candidate pathways for future functional validation regarding pregnancy-associated shifts in psoriasis pathology.

Monocytes/macrophages play a pivotal role in modulating the local immune environment of psoriasis. In our scRNA-seq analysis, these cells exhibited a tendency toward M2-like transcriptional signatures, characterized by high expression of M2 signature genes (e.g., *Mrc1*, *Arg1*, *Cd163*). Notably, cell–cell interaction analysis underscores the critical importance of predicted transcriptomic crosstalk between fibroblasts and monocytes/macrophages via the predicted GRN, and Gas6 communication networks (Figure S6A–C). Furthermore, we detected elevated expression of *Entpd1* (encoding CD39) in monocyte/macrophage cluster 1—a gene previously reported to be downregulated in Tregs within psoriasis lesional skin. Accumulating evidence has shown that CD39^+^ macrophages suppress the IL-23/Th17 axis in neutrophilic asthma and attenuate sepsis-induced liver injury by constraining pro-inflammatory signaling via the ATP-P2X7 receptor axis [65,66]. However, the specific pathways linking CD39-expressing monocytes/macrophages to attenuated psoriatic transcriptomic profiles remain to be fully elucidated, warranting further investigation.

Despite providing insights into pregnancy-associated regulation of psoriasis, this study has several limitations. First, our investigations relied solely on the IMQ-induced mouse model due to the clinical and ethical complexities of obtaining skin biopsies from acute pregnant psoriatic patients, meaning verification in human tissues is currently lacking. Second, our sequencing sample size (n = 3 per group) represents a standard benchmark for high-throughput profiling efficiency but restricts comprehensive multi-animal variance evaluation. Finally, while we experimentally confirmed hormone-induced secretion of PGRN and Gas6 by primary fibroblasts and documented in situ upregulation of macrophage MerTK, direct functional perturbation assays were not performed in this study. Specifically, testing whether conditioned medium from hormone-primed fibroblasts drives phenotypic skewing in bone marrow-derived macrophages (BMDMs) in a Gas6- or PGRN-dependent manner (using specific neutralizing antibodies or receptor antagonists) will be required to functionally establish this intercellular circuit. Furthermore, whether stromal TGF-β participates in disease attenuation in vivo via non-fibroblast sources or extracellular matrix-bound activation warrants subsequent investigation. Despite these limitations, our study advances the current understanding of how pregnancy-induced hormonal fluctuations modulate psoriasis and uncovers potential novel roles of fibroblasts, keratinocytes, and monocytes/macrophages in psoriatic pathogenesis. These findings provide insight for further exploration of pregnancy-associated immunomodulation in psoriasis.

In conclusion, through integrated bulk and scRNA-seq analyses, our study provides a detailed single-cell characterization of the effects of pregnancy on the IMQ-induced psoriatic dermatitis model. We identify fibroblast transcriptomic states as potential correlates of pregnancy-associated shifts and describe the homeostatic transcriptomic phenotypes observed in keratinocytes and monocytes/macrophages, alongside an endocrine-responsive stromal–myeloid axis characterized by fibroblast-derived PGRN and Gas6 and myeloid MerTK expression. These findings offer further insights into the modulation of psoriasis during pregnancy and provide a transcriptomic resource that may facilitate future investigations into the complexities of skin immunophysiology.

## Data Availability

The datasets generated and analyzed during the current study are available in the ArrayExpress repository (E-MTAB-16501, E-MTAB-16503) at https://www.ebi.ac.uk/biostudies/arrayexpress/studies/E-MTAB-16501 for E-MTAB-16501 and https://www.ebi.ac.uk/biostudies/arrayexpress/studies/E-MTAB-16503 for E-MTAB-16503 (accessed on 26 July 2026).

## Supplementary Materials

The following supporting information can be downloaded at https://www.mdpi.com/article/10.3390/biomedicines14092048/s1, Table S1: Detailed per-sample post-quality-control metrics. Figure S1: The top 10 pathways from KEGG enrichment analysis of downregulated DEGs (A) and upregulated DEGs (B), sorted by GeneRatio. Figure S2: The top 10 terms from GO enrichment analysis of upregulated DEGs (A) and downregulated DEGs (B), sorted by GeneRatio. Figure S3: (A) Average expression and expression percentages of lineage-specific markers in different cell types. (B) Differential number of intercellular interactions and differential intercellular communication strength between IMQ group and IMQ + pregnancy group. Figure S4: Comparison of the overall information flows of each signaling pathway between IMQ group and IMQ + pregnancy group; the top predicted information flows colored red were enriched in the IMQ group, and these colored green were enriched in the IMQ + pregnancy group. Figure S5: (A) Average expression and expression percentages of hormone-associated genes in different cell types. (B) The top 20 terms from GO enrichment analysis of upregulated and downregulated DEGs of fibroblasts, sorted by GeneRatio. (C) UMAP visualization of expression levels of hormone-associated genes in fibroblasts. Figure S6: Comparison of the overall information flows of each signaling pathway between IMQ group and IMQ + pregnancy group in fibroblasts; the top predicted information flows colored red were enriched in IMQ group, and these colored green were enriched in the IMQ + pregnancy group. Figure S7: Comparison of the overall information flows of each signaling pathway between IMQ group and IMQ + pregnancy group in monocytes/macrophages; the top predicted information flows colored red were enriched in the IMQ group, and these colored green were enriched in the IMQ + pregnancy group. Figure S8: Heatmap exhibiting the communication probabilities of TGF-β (A), GRN (B), and Gas6 (C) pathways in IMQ and the IMQ + pregnancy group. Figure S9: Heatmaps of DEGs for fibroblasts (A), monocytes/macrophages (B), spinosum keratinocytes (C), and basale keratinocytes (D). Data are clustered by DEGs. Figure S10: Comparison of the overall information flows of each signaling pathway between the IMQ group and IMQ + pregnancy group in stratum basale keratinocytes; the top predicted information flows colored red were enriched in the IMQ group, and those colored green were enriched in the IMQ + pregnancy group. Figure S11: Comparison of the overall information flows of each signaling pathway between IMQ group and IMQ + pregnancy group in stratum spinosum keratinocytes; the top predicted information flows colored red were enriched in IMQ group, and these colored green were enriched in the IMQ + pregnancy group. Figure S12: ELISA quantification of PGRN (A) and Gas6 (B) in cell culture supernatants after primary mouse dermal fibroblasts stimulated with 17β-estradiol, progesterone or growth hormone separately for 48 h. The cell experiments were repeated 3 times; * *p* < 0.05, ** *p* < 0.01, *** *p* < 0.001. Figure S13: Representative mIF images of lesional skin tissues from IMQ group. The red fluorescence represents F4/80, and the green fluorescence represents MerTK; bar = 100 μm. Figure S14: Representative mIF images of lesional skin tissues from the IMQ + pregnancy group. The red fluorescence represents F4/80, and the green fluorescence represents MerTK; bar = 100 μm. Figure S15: Quantification of the proportion of MerTK^+^ cells among F4/80^+^ cells; * *p* < 0.05, ** *p* < 0.01, *** *p* < 0.001.

## Abbreviations

The following abbreviations are used in this manuscript:

ANGPTL	Angiopoietin-Like Protein
ANOVA	Analysis of Variance
ARRIVE	Animal Research: Reporting of In Vivo Experiments
AVMA	American Veterinary Medical Association
cDNA	Complementary DNA
CD	Cluster of Differentiation (e.g., CD4, CD39, CD48, CD86, CD163, CD200)
DDRTree	Discriminative Dimensionality Reduction with Trees
DEG	Differentially Expressed Gene
E0/E8/E10/E15	Embryonic Day 0/8/10/15
Esr1	Estrogen Receptor 1
FC	Fold Change
FDR	False Discovery Rate
FGF	Fibroblast Growth Factor (e.g., FGF7, FGF10)
FPKM	Fragments Per Kilobase of Transcript per Million Mapped Reads
Gas6	Growth Arrest-Specific 6
Ghr	Growth Hormone Receptor
GO	Gene Ontology
Greb1	Growth Regulation by Estrogen in Breast Cancer 1
GRN/PGRN	Granulin Precursor/Progranulin
GSEA	Gene Set Enrichment Analysis
H&E	Hematoxylin and Eosin
IFN-γ	Interferon-Gamma
IL	Interleukin (e.g., IL-1b, IL-6, IL-10, IL-17, IL-22, IL-23, IL-34)
IMQ	Imiquimod
JAK	Janus Kinase
KEGG	Kyoto Encyclopedia of Genes and Genomes
Mif/MIF	Macrophage Migration Inhibitory Factor
mPASI	modified Psoriasis Area and Severity Index
Mertk	MER Proto-Oncogene, Tyrosine Kinase
NK cell	Natural Killer Cell
Oxtr	Oxytocin Receptor
PC	Principal Component
PCA	Principal Component Analysis
PD-1	Programmed Cell Death Protein 1
PD-L1	Programmed Death-Ligand 1
Pgr	Progesterone Receptor
Prlr	Prolactin Receptor
QC	Quality Control
RNA-seq	RNA Sequencing
scRNA-seq	Single-Cell RNA Sequencing
SEM	Standard Error of the Mean
Sema3	Semaphorin 3 (e.g., Sema3c, Sema3e)
SPF	Specific Pathogen-Free
STAT1	Signal Transducer and Activator of Transcription 1
TAM	Tyro3, Axl, and MerTK Receptor Family
TGF-β	Transforming Growth Factor-Beta
Th	T Helper Cell (e.g., Th1, Th2, Th17)
THBS	Thrombospondin
TNF-α	Tumor Necrosis Factor-Alpha
Treg	Regulatory T Cell
UMI	Unique Molecular Identifier
UMAP	Uniform Manifold Approximation and Projection
VISTA/Vsir	V-Set Immunoregulatory Receptor
