# Supplementary material for: Multi-Omics Analysis Identifies Fibroblast-Associated Stromal–Immune Networks Linked to Pregnancy-Associated Attenuation of Imiquimod-Induced Psoriatic Dermatitis
Source: Biomedicines. 2026 Sep 11;14(9):2048. doi: 10.3390/biomedicines14092048 (PMC13604052; doi:10.3390/biomedicines14092048)
Supplement: Supplementary file 1 [file biomedicines-14-02048-s001.zip › suplement figure.pdf]

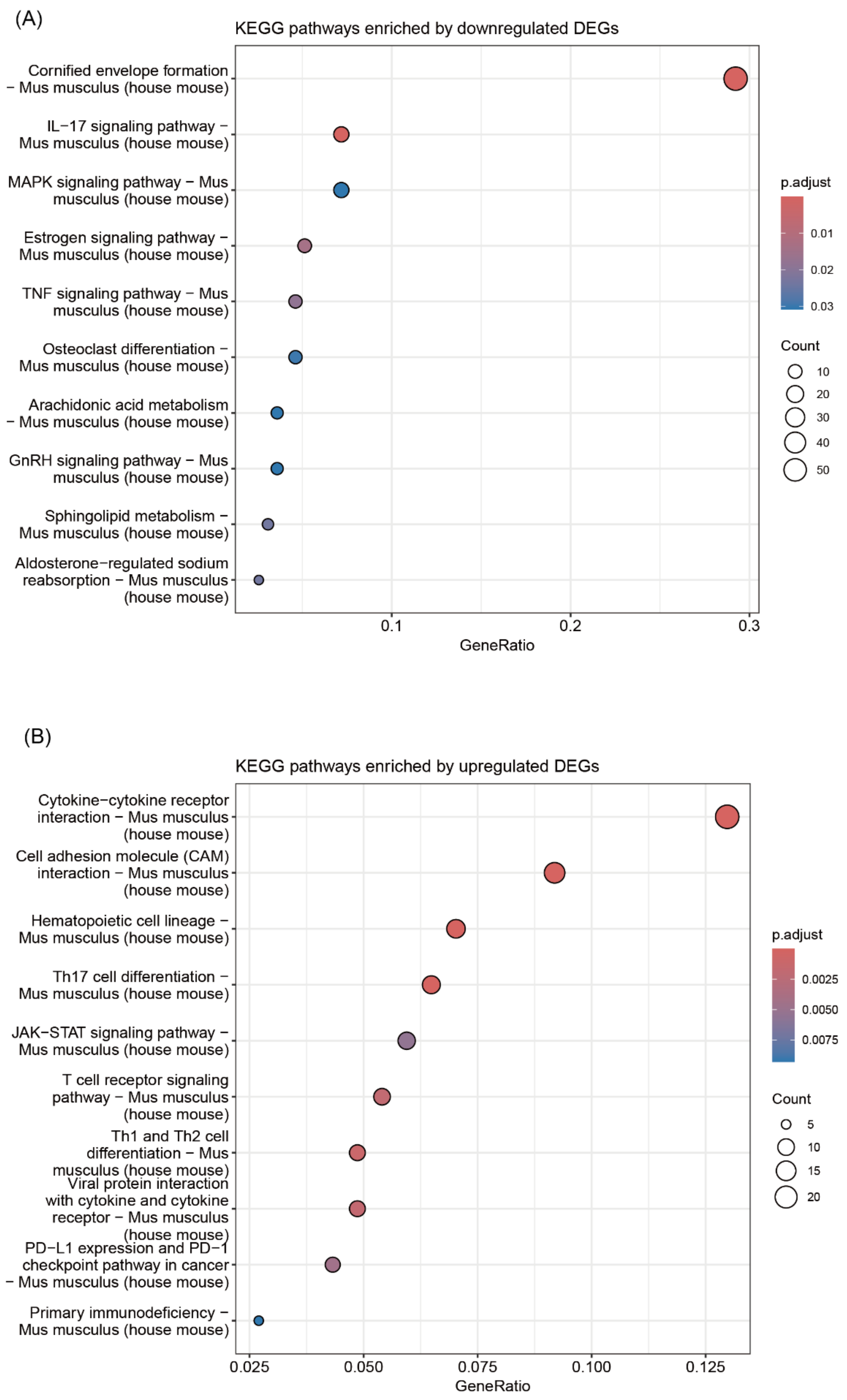

Figure S1

The top 10 pathways from KEGG enrichment analysis of downregulated DEGs **(A)** and upregulated DEGs **(B)**, sorted by GeneRatio.

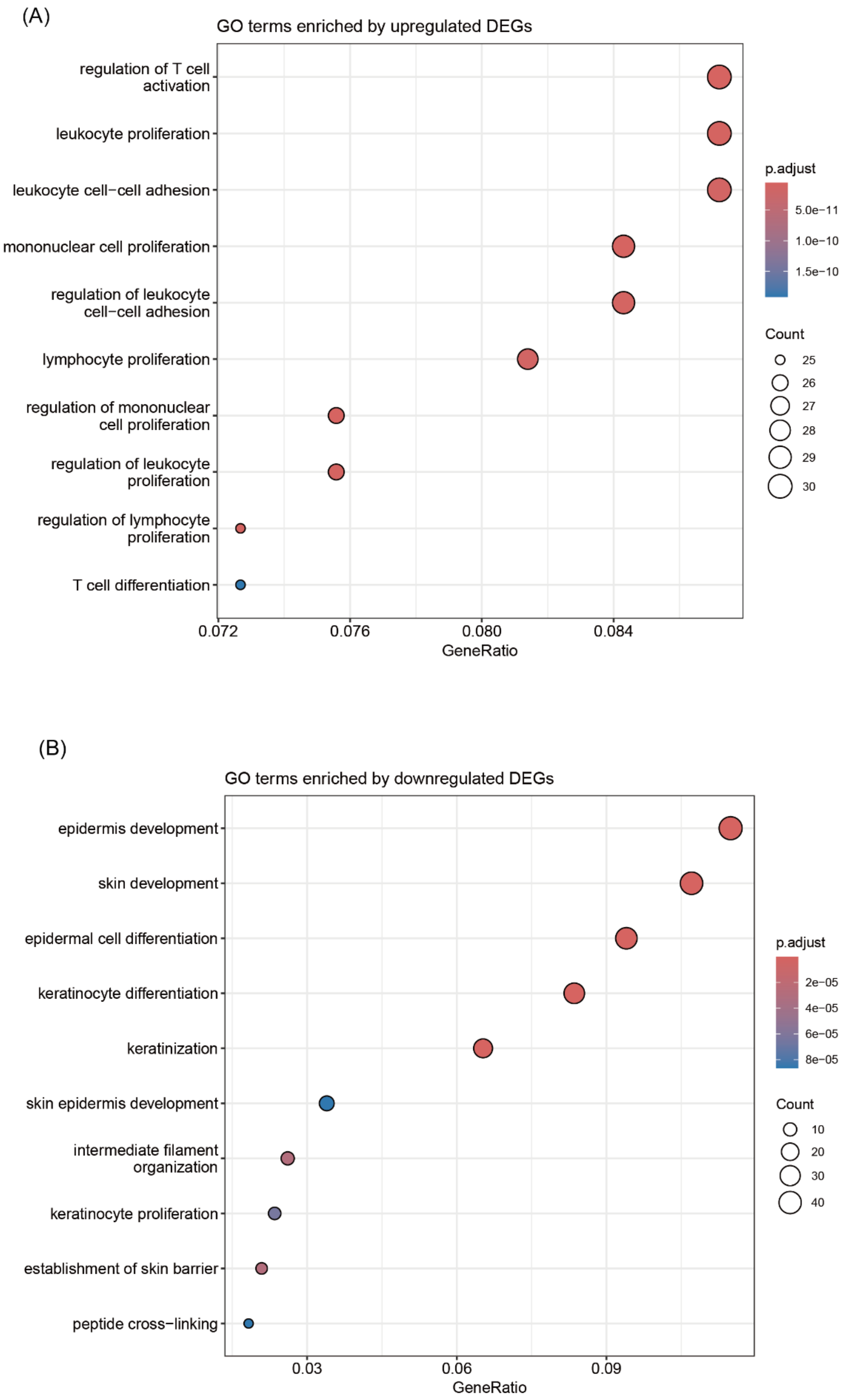

Figure S2

The top 10 terms from GO enrichment analysis of upregulated DEGs **(A)** and downregulated DEGs **(B)**, sorted by GeneRatio.

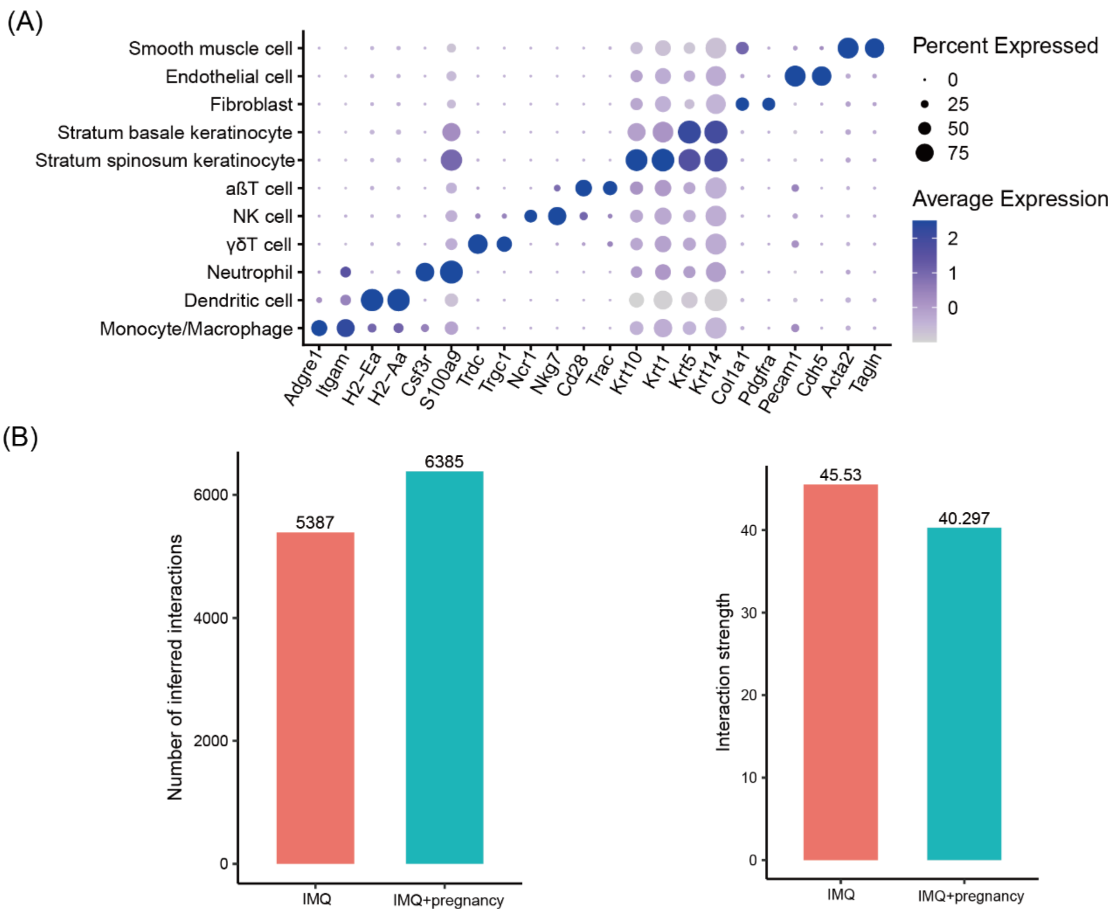

Figure S3

(A) Average expression and expression percentages of lineage-specific markers in different cell types. **(B)** Differential number of intercellular interactions and differential intercellular communication strength between IMQ group and IMQ + pregnancy group.

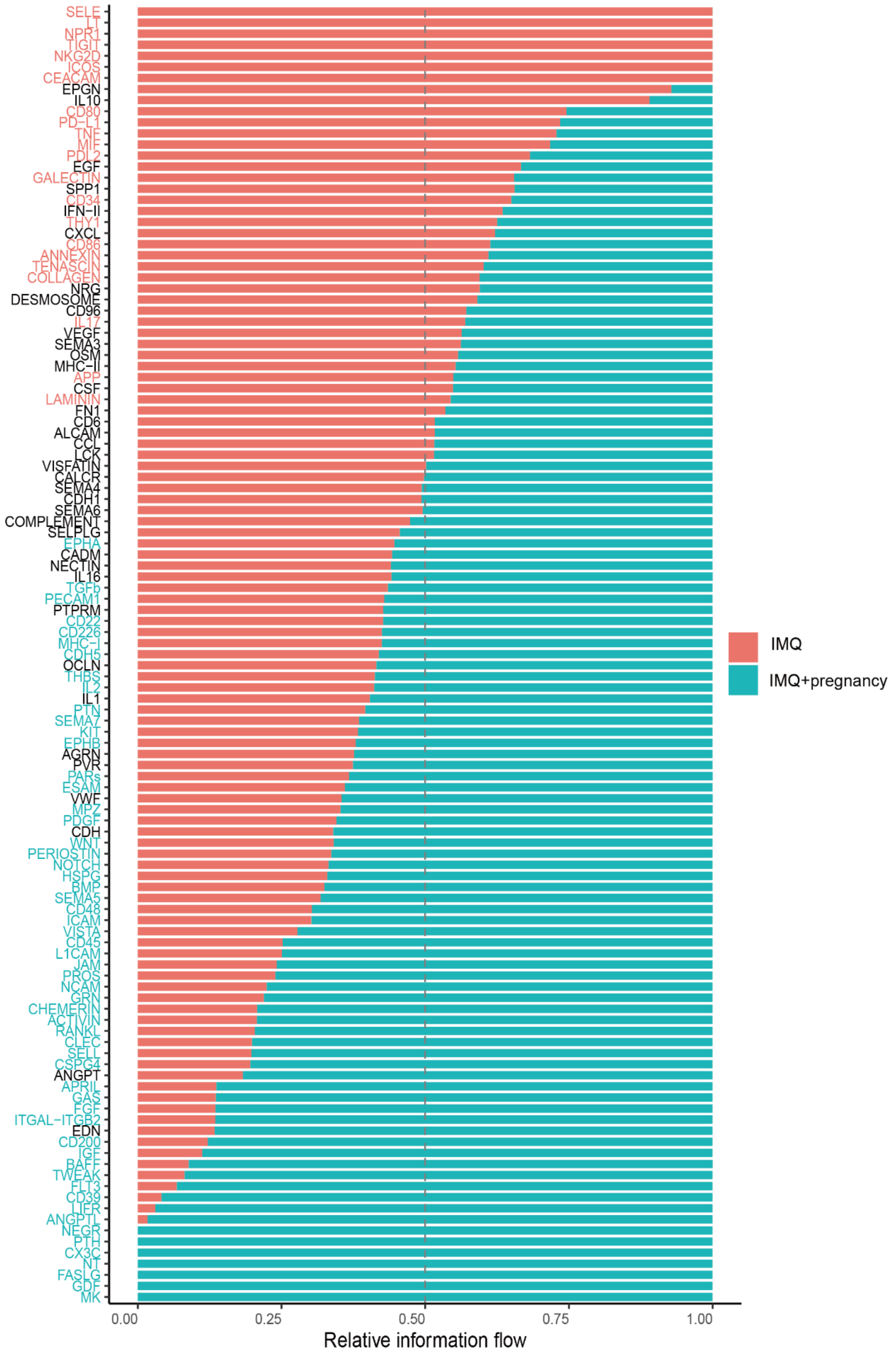

Figure S4

Comparison of the overall information flows of each signaling pathway between IMQ group and IMQ + pregnancy group; the top predicted information flows colored red

were enriched in IMQ group, and these colored green were enriched in the IMQ + pregnancy group.

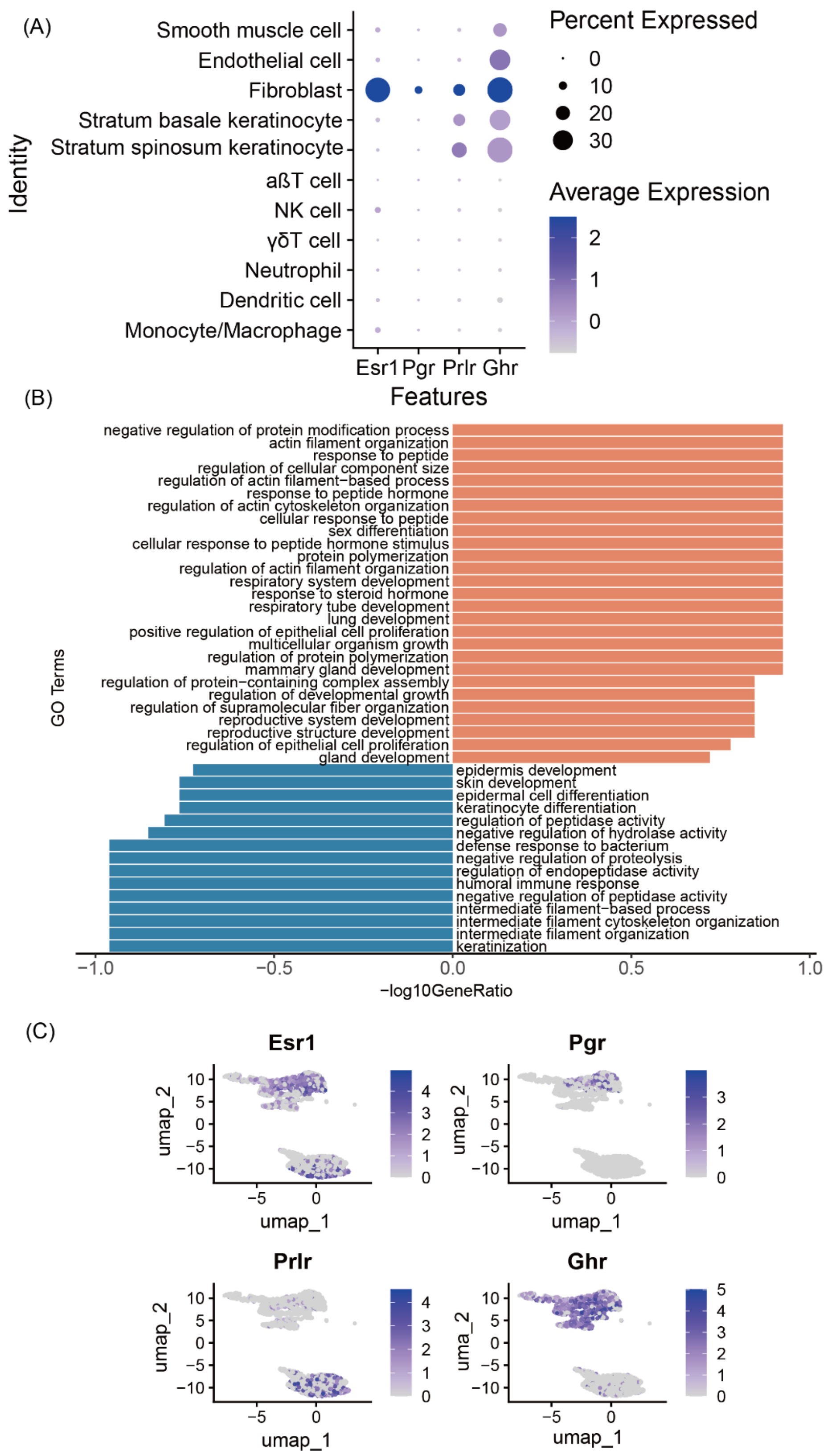

Figure S5

(A) Average expression and expression percentages of hormone-associated genes in different cell types. **(B)** The top 20 terms from GO enrichment analysis of upregulated

and downregulated DEGs of fibroblasts, sorted by GeneRatio. **(C)** UMAP visualization of expression levels of hormone-associated genes in fibroblast.

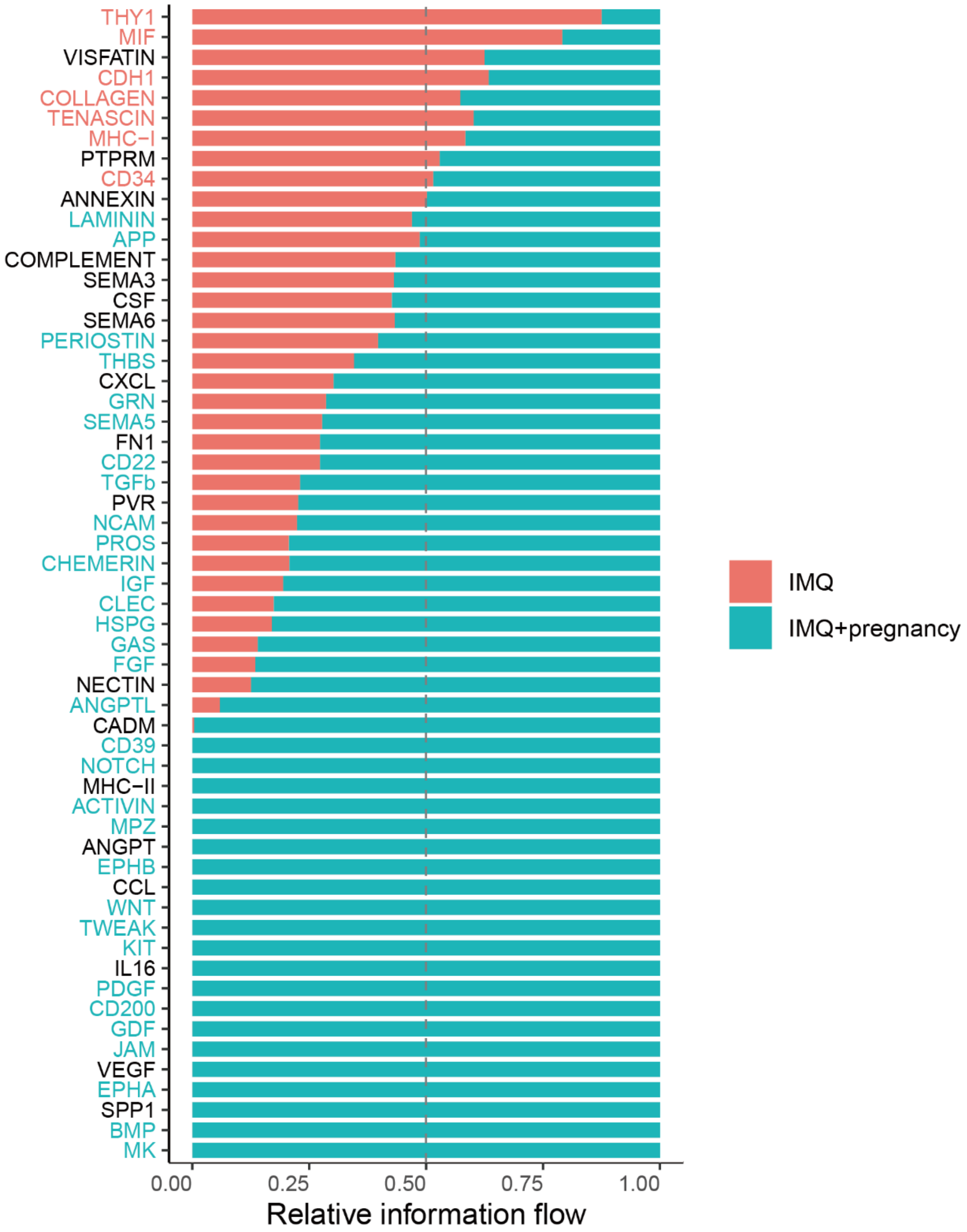

Figure S6

Comparison of the overall information flows of each signaling pathway between IMQ group and IMQ + pregnancy group in fibroblasts; the top predicted information flows colored red were enriched in IMQ group, and these colored green were enriched in the IMQ + pregnancy group.

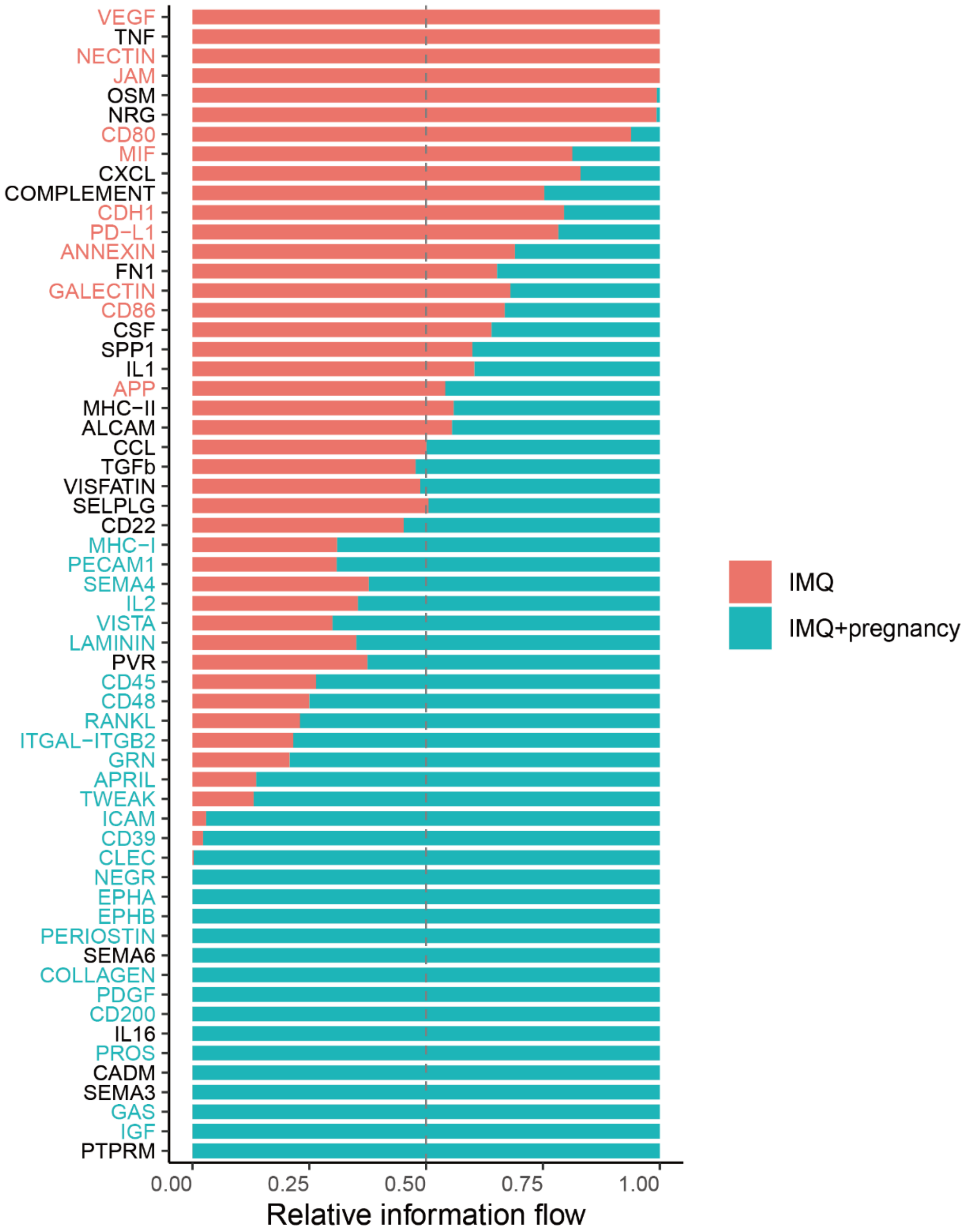

Figure S7

Comparison of the overall information flows of each signaling pathway between IMQ group and IMQ + pregnancy group in monocytes/macrophages; the top predicted information flows colored red were enriched in IMQ group, and these colored green were enriched in the IMQ + pregnancy group.

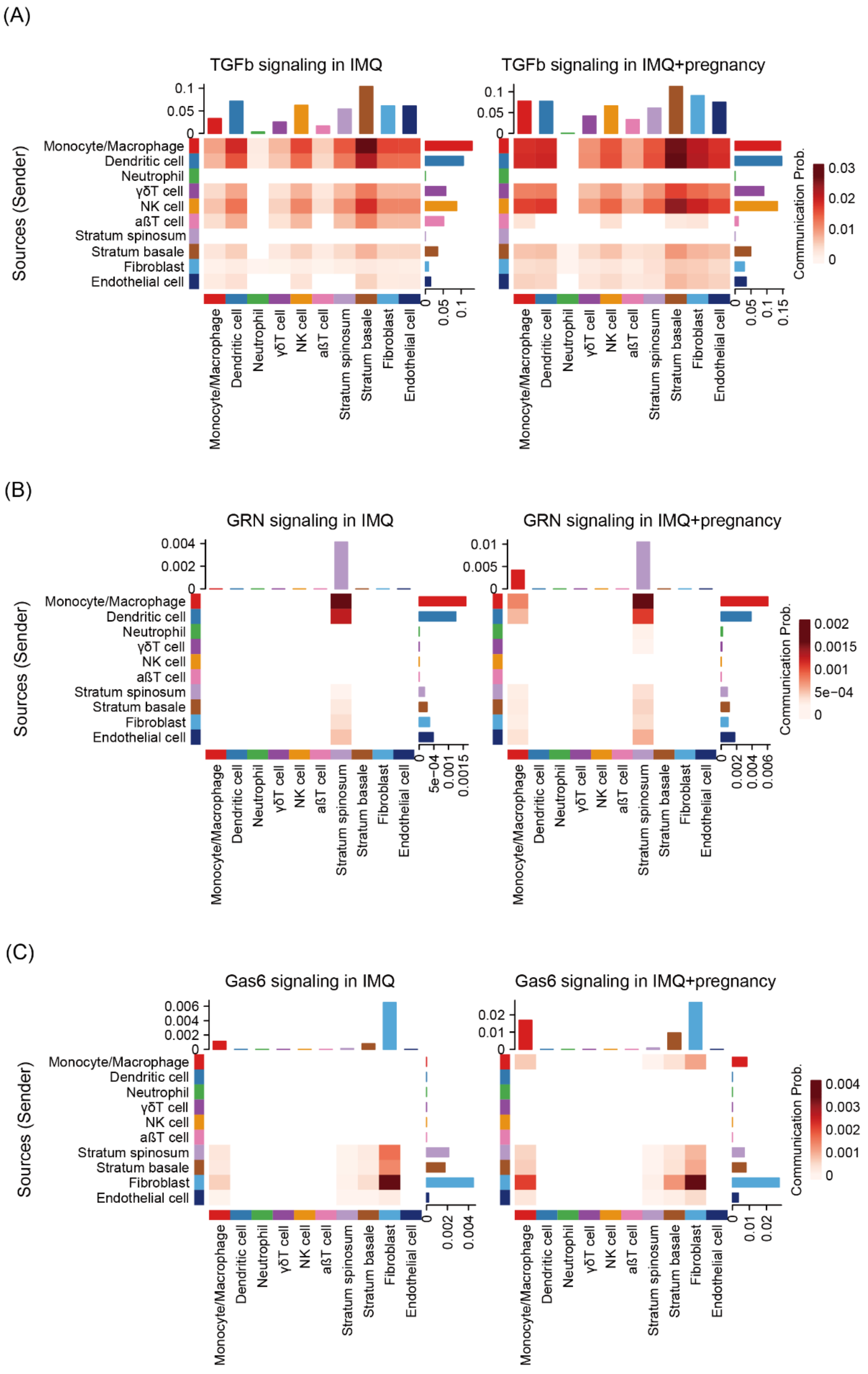

Figure S8

Heatmap exhibit the communication probability of TGF-β **(A)**, GRN **(B)**, Gas6 **(C)** pathways in IMQ and IMQ + pregnancy group.

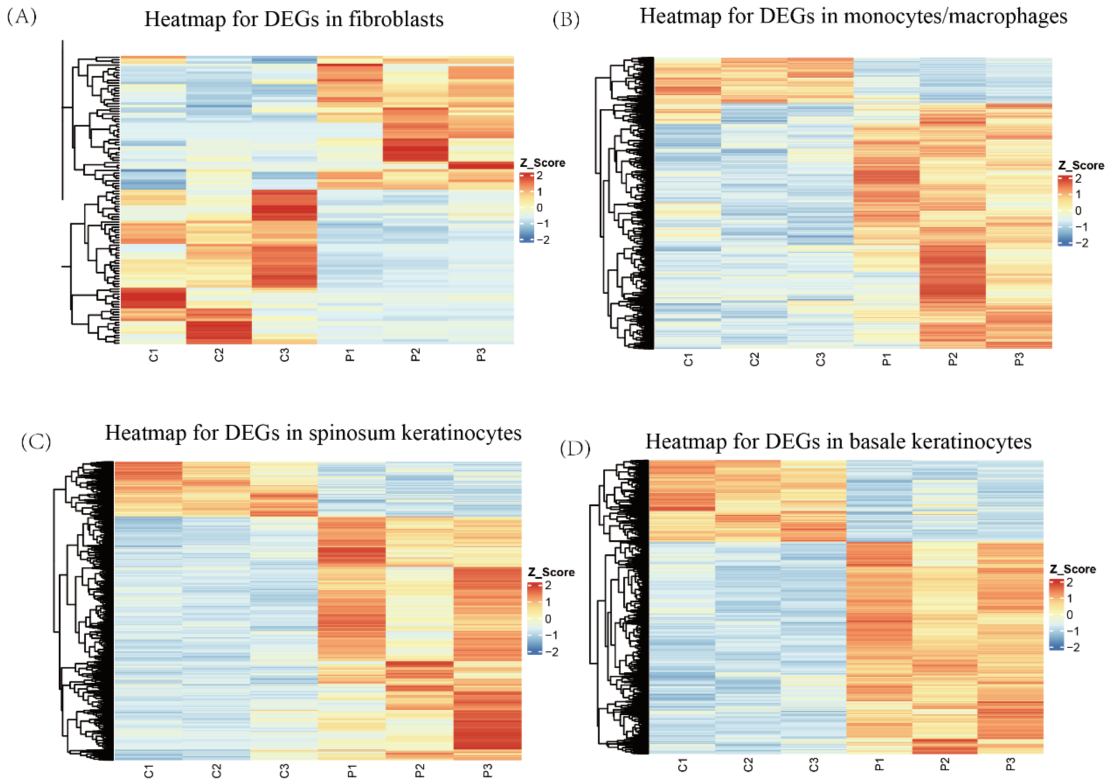

Figure S9

Heatmaps of DEGs for fibroblasts (A), monocytes/macrophages (B), spinosum keratinocytes (C), and basale keratinocytes (D). Data are clustered by DEGs.

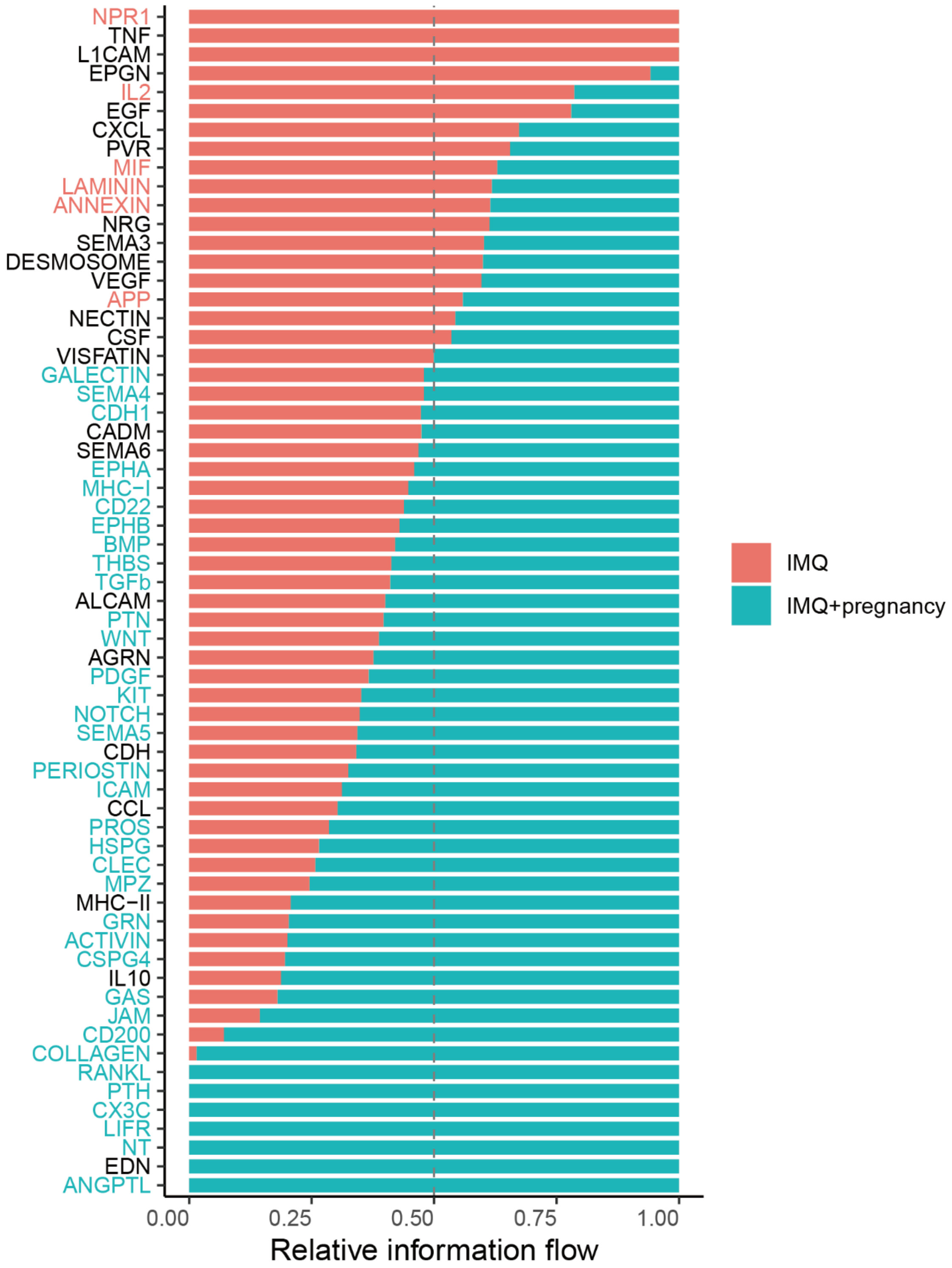

Figure S10

Comparison of the overall information flows of each signaling pathway between IMQ group and IMQ + pregnancy group in stratum basale keratinocytes; the top predicted information flows colored red were enriched in IMQ group, and these colored green were enriched in the IMQ + pregnancy group.

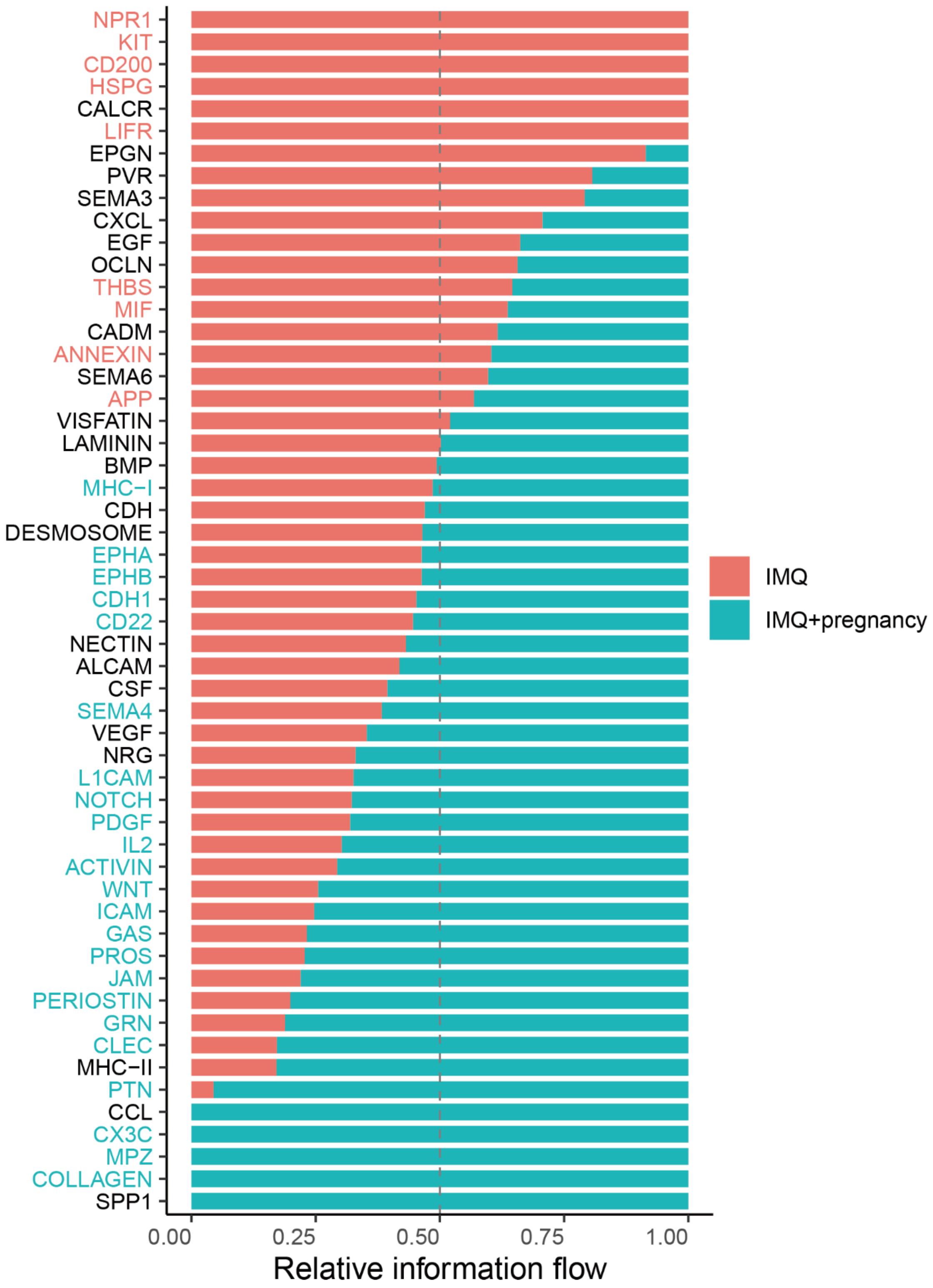

Figure S11

Comparison of the overall information flows of each signaling pathway between IMQ group and IMQ + pregnancy group in stratum spinosum keratinocytes; the top predicted information flows colored red were enriched in IMQ group, and these colored green were enriched in the IMQ + pregnancy group.

(A)

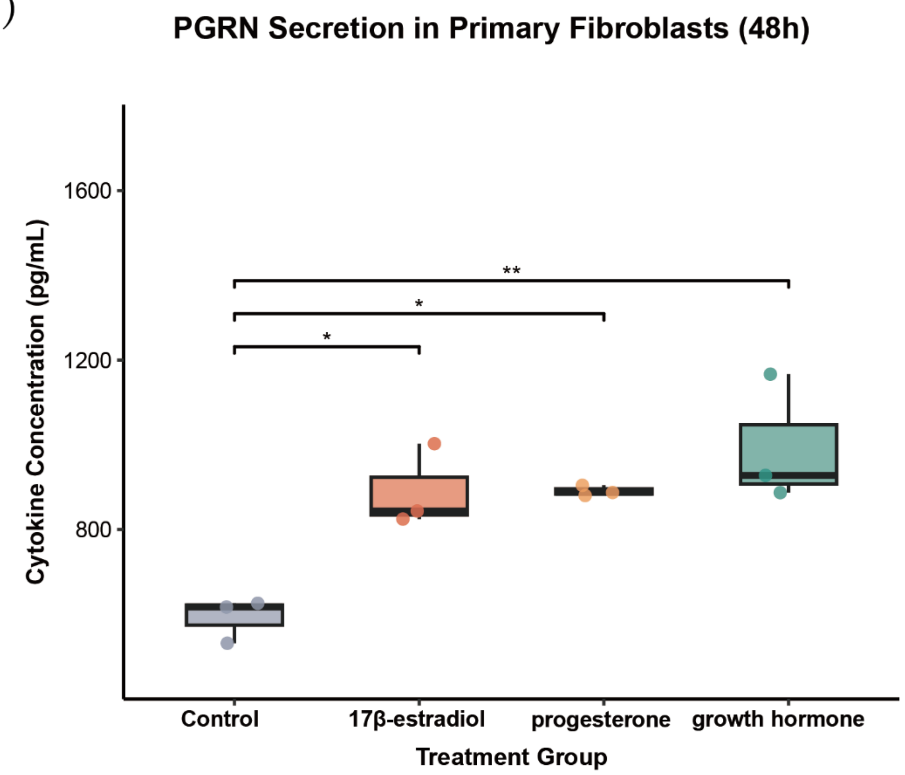
PGRN Secretion in Primary Fibroblasts (48h)
Cytokine Concentration (pg/mL)
1600
1200
800
**
*
*
Control
17β-estradiol
progesterone
growth hormone
Treatment Group

(B)

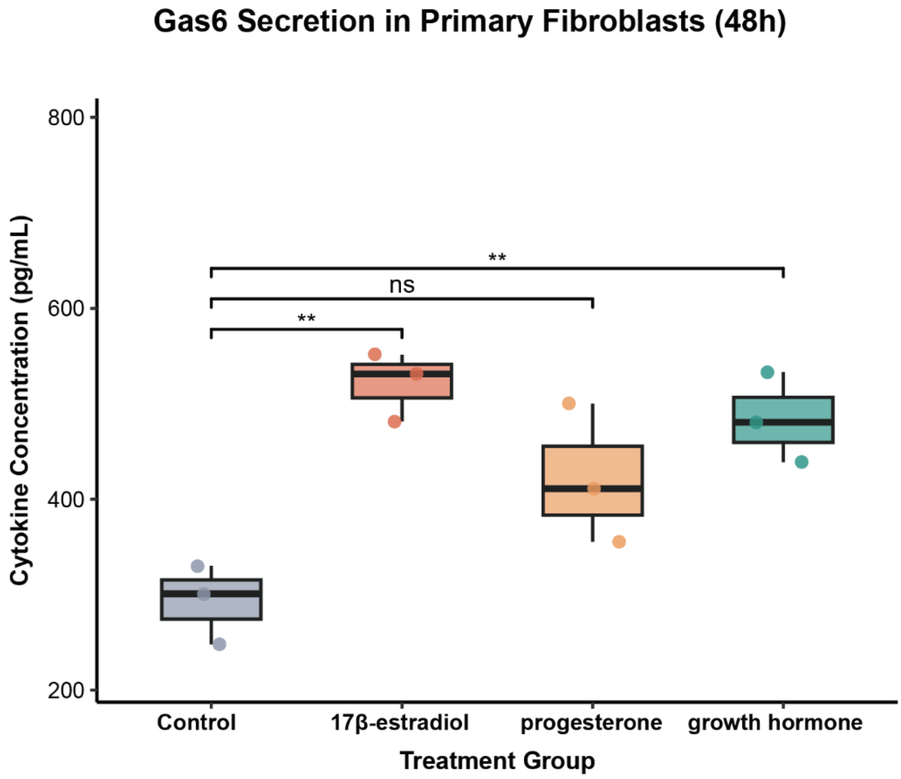
Gas6 Secretion in Primary Fibroblasts (48h)
Cytokine Concentration (pg/mL)
800
600
400
200
**
ns
**
Control
17β-estradiol
progesterone
growth hormone
Treatment Group

Figure S12

ELISA quantification of PGRN (A) and Gas6 (B) in cell culture supernatants after primary mouse dermal fibroblasts stimulated with 17β-estradiol, progesterone or growth hormone separately for 48 hours; The cell experiments were repeated 3 times; * $p < 0.05$, ** $p < 0.01$, *** $p < 0.001$.

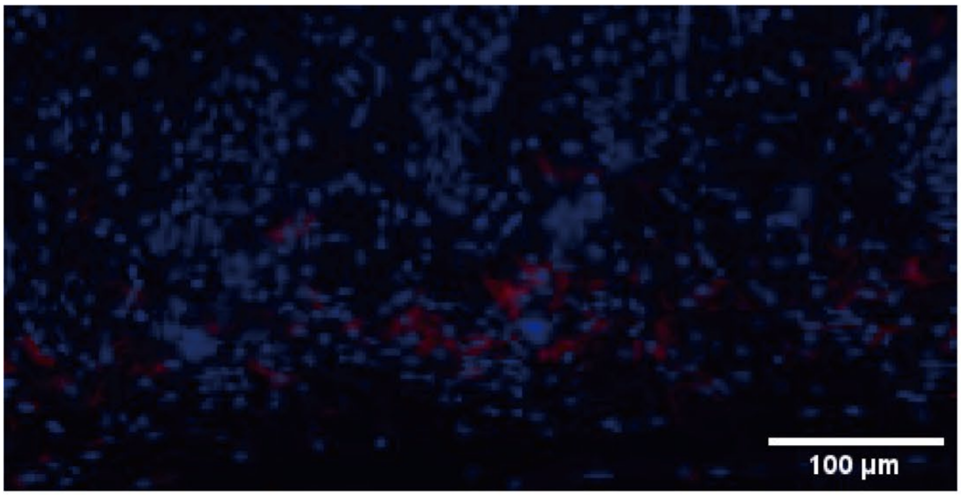

F4/80

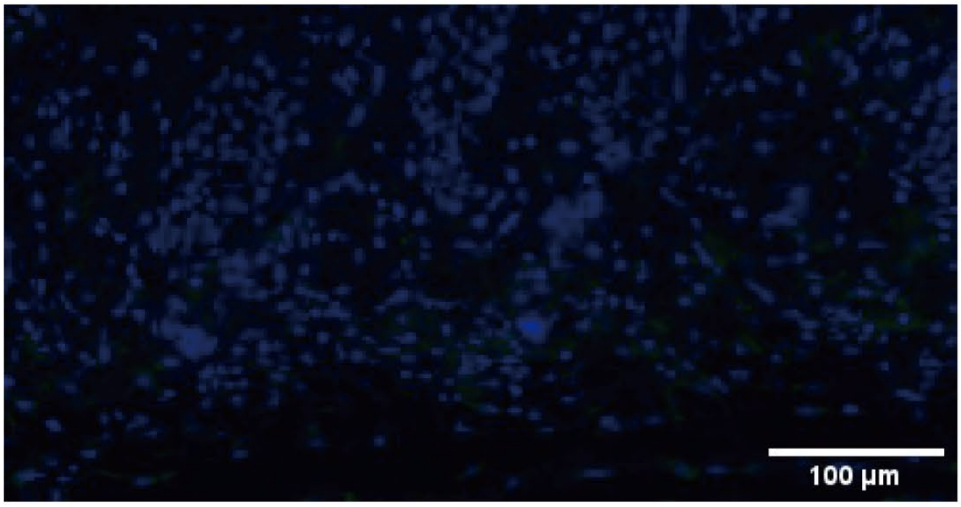

MerTK

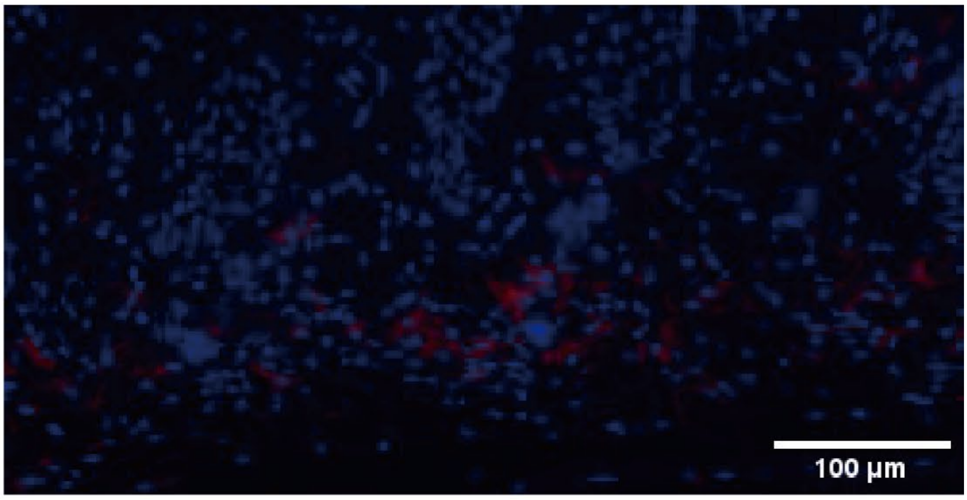

Merge

Figure S13

Representative mIF images of lesional skin tissues from IMQ group. The red fluorescence represents for F4/80, and the green fluorescence represents for MerTK; Bar = 100μm.

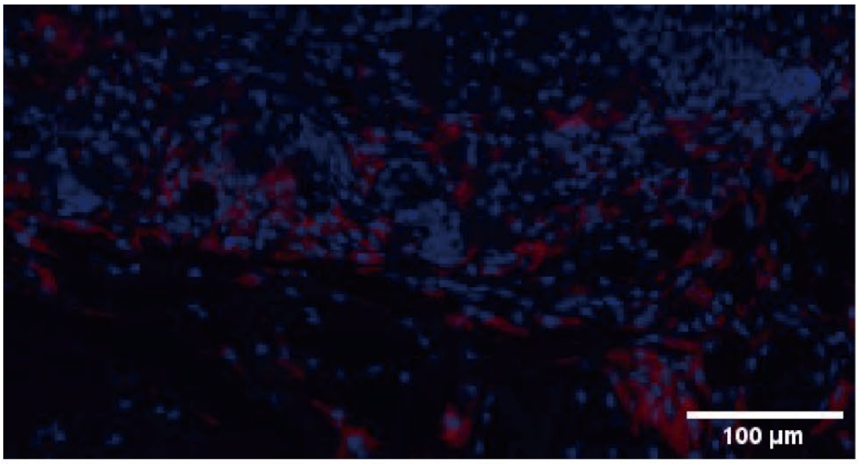

F4/80

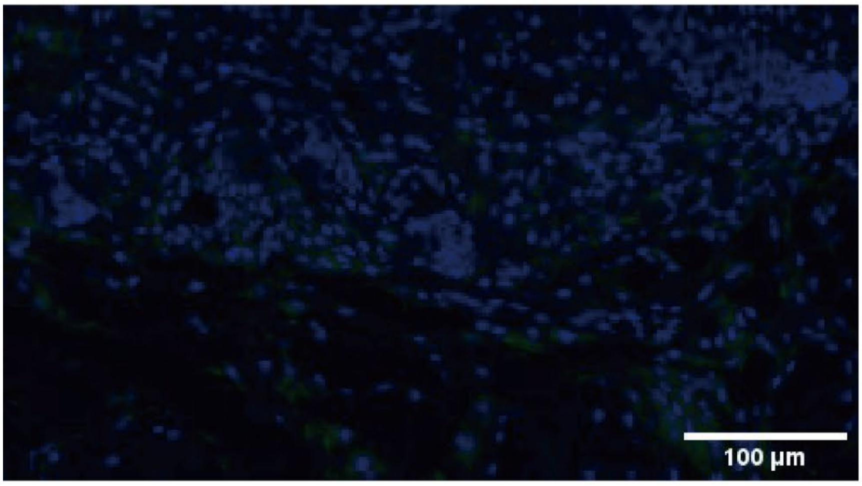

MerTK

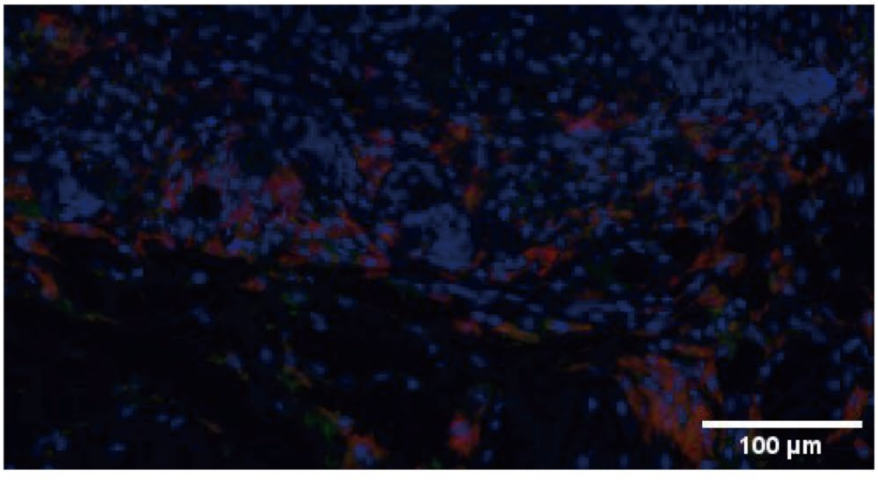

Merge

Figure S14

Representative mIF images of lesional skin tissues from IMQ + pregnancy group. The red fluorescence represents for F4/80, and the green fluorescence represents for MerTK; Bar = 100μm.

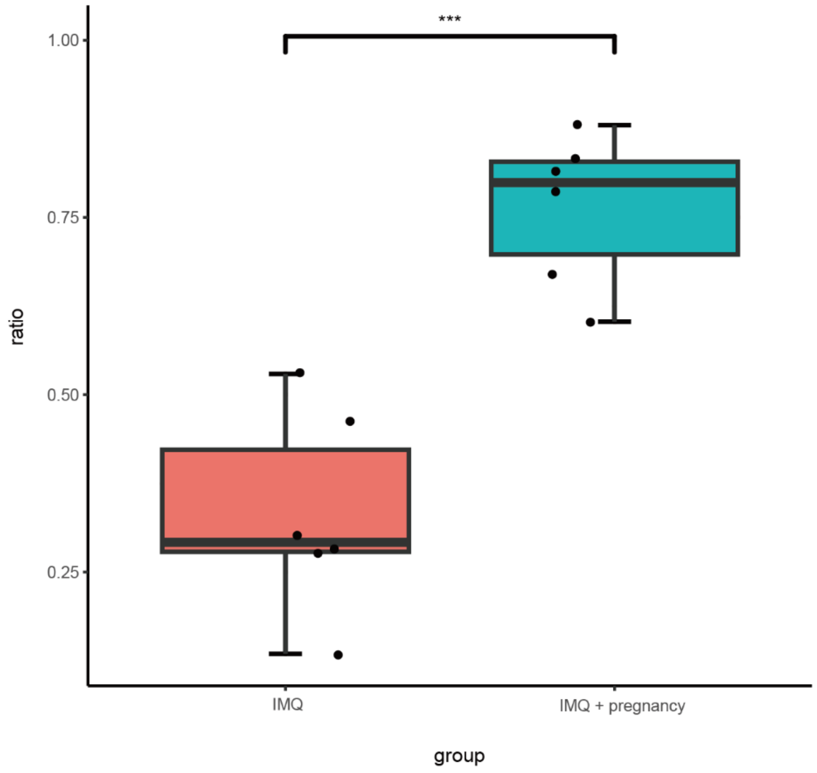

Figure S15

Quantification of the proportion of MerTK$^+$ cells among F4/80$^+$ cells; * $p < 0.05$, ** $p < 0.01$, *** $p < 0.001$.; * $p < 0.05$, ** $p < 0.01$, *** $p < 0.001$.
